# Identifying Receptor Kinase Substrates Using an 8000 Peptide Kinase Client Library Enriched for Conserved Phosphorylation Sites

**DOI:** 10.1016/j.mcpro.2025.100926

**Published:** 2025-02-07

**Authors:** Daewon Kim, Gabriel Lemes Jorge, Chunhui Xu, Lingtao Su, Sung-Hwan Cho, Nagib Ahsan, Dongqin Chen, Lijuan Zhou, Marina A. Gritsenko, Mowei Zhou, Jinrong Wan, Ljiljana Pasa-Tolic, Dong Xu, Laura E. Bartley, Jay J. Thelen, Gary Stacey

**Affiliations:** 1 Division of Plant Science & Technology, C.S. Bond Life Sciences Center, University of Missouri, Columbia, Missouri, USA; 2 Division of Biochemistry and Interdisciplinary Plant Group, C.S. Bond Life Sciences Center, University of Missouri, Columbia, Missouri, USA; 3 Department of Electrical Engineering and Computer Science, C.S. Bond Life Sciences Center, Institute for Data Science and Informatics, University of Missouri, Columbia, Missouri, USA; 4 Department of Chemistry and Biochemistry, The University of Oklahoma, Norman, Oklahoma, USA; 5 Mass Spectrometry, Proteomics and Metabolomics Core Facility, Stephenson Life Sciences Research Center, University of Oklahoma, Norman, Oklahoma, USA; 6 Environmental Molecular Sciences Laboratory, Pacific Northwest National Laboratory, Richland, Washington, USA; 7 Institute of Biological Chemistry, Washington State University, Pullman, Washington, USA

**Keywords:** Kinase-client assay, P2K1, receptor-like kinase, Arabidopsis thaliana, phosphopeptides

## Abstract

In eukaryotic organisms, protein kinases regulate diverse protein activities and signaling pathways through phosphorylation of specific protein substrates. Isolating and characterizing kinase substrates is vital for defining downstream signaling pathways. The kinase-client (KiC) assay is an *in vitro* synthetic peptide LC-MS/MS phosphorylation assay that has enabled identification of protein substrates (*i.e.*, clients) for various protein kinases. For example, previous use of a 2100-member (2k) peptide library identified substrates for the extracellular ATP receptor-like kinase, P2K1. Many P2K1 clients were confirmed by additional *in vitro* and *in planta* studies, including integrin-linked kinase 4, for which we provide the evidence herein. In addition, we developed a new KiC peptide library containing 8000 (8k) peptides based on phosphorylation sites primarily from *Arabidopsis thaliana* datasets. The 8k peptides are enriched for sites with conservation in other angiosperm plants, with the paired goals of representing functionally conserved sites and usefulness for screening kinases from diverse plants. Screening the 8k library with the active P2K1 kinase domain identified 177 phosphopeptides, including calcineurin B–like protein and G protein alpha subunit 1, which functions in cellular calcium signaling. We confirmed that P2K1 directly phosphorylates calcineurin B–like protein and G protein alpha subunit 1 through *in vitro* kinase assays. This expanded 8k KiC assay will be a useful tool for identifying novel substrates across diverse plant protein kinases, ultimately facilitating the exploration of previously undiscovered signaling pathways.

## Highlights

• A 2k peptide library identified ILK4 as a P2K1 kinase substrate in the KiC assay.
• An 8k peptide library was built using known Arabidopsis phosphorylation sites and conserved motifs.
• Using the expanded KiC library, 177 phosphopeptides with 185 phosphorylation sites were identified as substrates of P2K1.
• The interaction and phosphorylation of CBL9 and GPA1 by P2K1 receptor-like kinase were verified using split-LCI, BiFC, and *in vitro* kinase assays.

## Graphical Abstract

**Figure:**
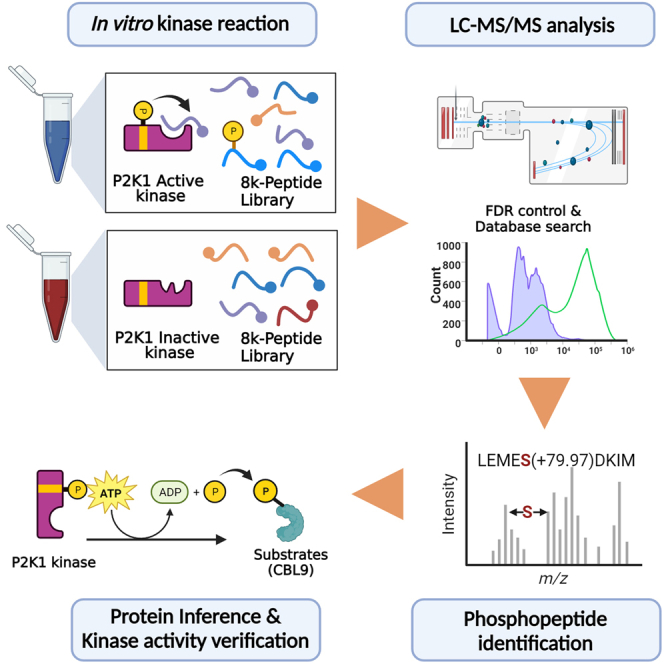

## In Brief

The Kinase Client (KiC) assay is an essential tool for identifying novel protein kinase substrates. Using an expanded 8k peptide library enriched for conserved and potentially conserved peptides phosphorylated in angiosperm plants, we identified 177 peptides (185 residues) phosphorylated by P2K1, a receptor-like kinase for extracellular ATP. Among the potential substrates, biochemical experiments support CBL9 and GPA1 as likely P2K1 clients. This expanded 8k KiC assay is a valuable resource for isolating novel substrates of plant protein kinases, facilitating the discovery of new substrate interactions.

Protein phosphorylation is a widespread posttranslational modification (PTM) that is crucial for regulating diverse cellular processes and signaling pathways in eukaryotic organisms (1, 2). Key regulators in these processes are enzymes known as protein kinases (1, 3). The genomes of many multicellular eukaryotes possess about ∼450 core protein kinases, or ∼2% of the genome, from well-known families such as cyclin-dependent protein kinases, calcium-dependent kinases, and mitogen-activated protein (MAP) kinases, among others. Receptor-like kinases (RLKs) serve as sentinels to detect extracellular signals and trigger changes intracellularly. In plants, the number of RLKs has expanded greatly relative to fungi and animals, with, for example, the genome of Arabidopsis encoding 615 RLKs, compared to 4 in humans (4). Indeed, approximately 41% of expressed Arabidopsis proteins undergo phosphorylation, primarily on serine (S), threonine (T), and tyrosine (Y) amino acid residues (3, 5). Thus, protein kinase–mediated signaling appears to be particularly important for plant processes and reliable and facile approaches to interrogate these signaling cascades remain of crucial importance for understanding molecular mechanisms of development and interactions with the environment.

In an *in vitro* kinase assay, a purified kinase enzyme is incubated with a potential substrate molecule in the presence of ATP, which serves as the phosphate donor (6). The kinase catalyzes transfer of a phosphate group from ATP to the substrate molecule. The phosphorylation of the substrate can be detected and quantified through various methods, such as autoradiography, immunoblotting with phospho-specific antibodies, or mass spectrometry (6). This approach is widely employed to study kinase–substrate interactions, assess kinase activity, and investigate signal transduction pathways in a controlled experiment. However, discovering new kinase protein substrates through *in vitro* assays can be challenging (6, 7).

In previous research, a novel methodology was reported that combines the ease of *in vitro* kinase profiling with the precision of mass spectrometry–based detection of phosphopeptides (7, 8). This approach, termed the kinase-client (KiC) assay, expedites high-throughput exploration of kinase-substrate interactions (7, 8). The KiC assay workflow consists of four stages. First, active protein kinases are purified and a synthetic peptide library is constructed. Second, the kinase and peptide library(-ies) are mixed in an *in vitro* kinase assay. Third, peptides are separated and analyzed *via* LC-MS/MS analysis. Fourth, the data are analyzed through spectral counting and contextualized through subsequent database searches. The KiC assay capitalizes on solution-phase peptide libraries, offering scalability advantages over peptide chips. Furthermore, the LC-MS/MS succeeds in the separation and identification of phosphopeptides amidst a complex mixture of unphosphorylated peptides. This methodology eliminates the need for radioisotopes or phospho-specific antibodies for the detection of phosphopeptides (7, 8, 9), both of which suffer from nonspecific background signal.

KiC assay results have been effectively used in interrogating the extracellular ATP (eATP) signaling pathway of Arabidopsis. In both plants and animals, eATP serves as a damage-associated molecular pattern. Mammals possess two classes of eATP purinoreceptors, the ligand-gated ion channel, P2X, and the G protein–coupled receptor (GPCR), P2Y (10). These receptors respond to eATP and function in a variety of physiological processes, such as immune response, inflammation, neurotransmission, muscle contraction, and cell death (11, 12, 13). In addition to a role in plant stress responses, eATP plays a significant role in gravitropism, root hair growth, root avoidance, thigmotropism, and cell death (14, 15, 16, 17, 18, 19, 20, 21, 22, 23). However, homologs of the canonical P2X and P2Y purinoreceptors are absent in plants (24, 25). Instead, in Arabidopsis the P2K1 (DORN1/LecRK I.9) purinoreceptor, a lectin RLK, recognizes eATP released during growth and in response to a variety of abiotic and biotic stresses (24). The P2K1 receptor functions as a positive regulator of plant defense against various pathogens, including *Pseudomonas syringae* pv. tomato DC3000 (*Pst* DC3000), *Phytophthora infestans*, and *Phytophthora brassicae* (14, 26, 27, 28, 29). Arabidopsis also possesses a second purinoreceptor, P2K2 (LecRK I.5), which interacts with P2K1 and also plays a role in stress responses (14, 24, 30, 31, 32).

Previously, we used the P2K1 kinase domain to screen a 2100-member (2k) peptide KiC library and identified over 20 putative substrates for this kinase (14, 33, 34). Among these candidates was respiratory burst oxidase homolog D (RBOHD), NADPH oxidase, which we subsequently confirmed *in planta* to be a substrate of P2K1 showing that eATP is an important regulator of stomatal aperture (14). We also confirmed that protein acyltransferases, identified *via* the KiC assay, are P2K1 phosphorylation targets *in planta*, acting as negative regulators of P2K1 function (34). The KiC assay also identified integrin-linked kinase 5 (ILK5/Raf27/blue light–dependent H^+^-ATPase phosphorylation; hereafter referred to as ILK5; a MAP kinase kinase kinase) as a substrate of P2K1 phosphorylation with subsequent confirmation elucidating the role of ILK5 is mediating the activation of the MAP kinase pathway in response to eATP signaling (33, 35).

The initial 2k KiC peptide library was designed based on Arabidopsis phosphopeptide data available when plant phosphoproteomics was still relatively new. Though the KiC assay with the 2k library has been successfully utilized for protein kinases isolated from diverse plant species, including Ricinus and Arabidopsis (14, 33, 34, 36), in designing a new plant KiC library, we wanted to maximize the utility of the library by focusing on conserved or potentially conserved phosphosites. Among others, we also include Arabidopsis phosphopeptides that are expressed in roots, due to the importance of root processes in sustainable agriculture. As an initial test of this 8k KiC peptide library, we applied this library to expand the list of putative P2K1 kinase substrates.

## Experimental Procedures

### Experimental Design and Statistical Rationale

The main objective of this study is to identify putative substrates of the ATP RLK P2K1 protein using an extended 8K peptide-based KiC assay. To maximize the identification rate using data-dependent acquisition (DDA) acquisition mode, each 1000-peptide pool was injected separately. Typically, over 95% of the synthetic peptides in their pure form were detectable, with the majority showing charge states of 2+ and 3+, and occasionally reaching up to 6+. Data from negative control identification results, including Ascore, PTM ion intensity, and peptide spectrum match (PSM) counts, were used to develop effective filtration criteria for phosphopeptide candidate identification. For the split-luciferase imaging assay, four times (calcineurin B–like protein 9 [CBL9] or G protein alpha subunit 1 [GPA1]) or eight times (ILK4) biological replicates were performed on *Nicotiana benthamiana* leaves, and bioluminescence was measured using C-vision/Im32 software. The results are presented as mean ± SEM. Statistical significance of each value was determined using GraphPad (version 8; https://www.graphpad.com/scientific-software/prism/) with an unpaired two-tailed Student’s *t* test. Significance levels are denoted as follows: ∗∗∗∗*p* < 0.0001, ∗∗∗*p* < 0.001, ∗∗*p* < 0.01, and ∗*p* < 0.05. Quantification of phosphorylation signal intensities were captured using the Typhoon FLA 9000 Phospho-imager (GE Healthcare) and analyzed with ImageJ (https://imagej.net/ij/). The data are presented as mean ± SEM (n = 4). Statistical significance was calculated using GraphPad Prism software, with ∗∗*p* < 0.01. The *p* value indicates the level of significance, determined through an unpaired two-tailed Student’s *t* test, by comparing the band intensity to that of the control protein, glutathione-*S*-transferase (GST).

### Plant Materials and Growth Conditions

This study employed *A. thaliana* ecotype Columbia (Col-0) along with three T-DNA insertion mutants (WiscDsLox345-348B17; *ilk5-1*, Salk_152804C; *ilk4-1 Salk_042209; and p2k1-3*) and transgenic lines–expressing *ILK4promoter::*β-glucuronidase (*GUS*) and *ILK5promoter::GUS*, as well as *OXP2K1*, *P2K1-YFP*, *Free-YFP*, and *ILK4-YFP*. Seeds were surface-sterilized by immersion in a 70% (v/v) ethanol solution for 10 min, followed by five washes with sterile water. Subsequently, seeds were germinated on agar plates containing half-strength Murashige and Skoog medium supplemented with 1% (w/v) sucrose, 0.5% (w/v) phytagel, and 0.05% (w/v) MES, pH adjusted to approximately 5.7 with KOH. The seeds underwent 3 days of vernalization at 4 °C in darkness before being transferred to a growth chamber for germination. After achieving seedling growth, plants were transplanted into soil and cultivated under long-day conditions (16-h photoperiod with a light intensity of 150 μE m^−2^ s^−1^ provided by white LED lamps) at 21 °C until maturity for subsequent experiments.

### Plasmid Constructs and Site-Directed Mutagenesis

The full-length coding sequences of ILK4 (*At3g58760*), ILK5 (*At4g18950*), ILK6 (*At1g14000*), GPA1 (*At2g26300*), P2K1 (*At5g60300*), RBOHD (*At*5*g47910*), LYK5 (*At2g33580*), MKK3 (*At5g40440*), and MKK8 (*AT3G06230*), along with their kinase domain or C-terminal domain, were PCR-amplified using gene-specific primer pairs (Supplemental Table S1). The resulting PCR products from complementary DNA were subcloned into *pDONR-Zeo* (Invitrogen) or *pGEM-T* Easy vectors (Promega) for further experiments. To generate YFP constructs, the *pAM-PAT-GW-YFP* vector was used. For the bimolecular fluorescence complementation (BiFC) experiment in Arabidopsis protoplasts, the *pAM-PAT-GW-nYFP* and *pAM-PAT-GW-cYFP* gateway vectors were C terminally fused with split-YFP. For the split-luciferase complementation imaging (LCI) assays in *N. benthamiana* leaves, the full-length coding sequence DNAs from the *pDONR-Zeo* vector were cloned into the *pCAMBIA1300-GW-nLUC* and *pCAMBIA1300-GW-cLUC* gateway vectors. The *ILK4* and *ILK5* promoters were cloned as a 2 kb fragment upstream of the translation start codon into the *pDONR-Zeo* vector by BP reaction. *ILK4promoter::GUS* and *ILK5promoter::GUS* were generated by LR cloning into *pGWB3* (GUS) gateway vector. GST-fused P2K1, the kinase-dead version of P2K1 (GST-P2K1^D572N^-cytosolic domain [CD]) and LysM-containing RLK 5 (GST-LYK5-CD) were described previously (14, 33). To generate the GST-tagged CBL9 construct, DNA fragments of CBL9 were PCR-amplified using specific primers, digested with EcoRI/SalI and cloned into the *pGEX5X-1* (GE Healthcare) vector. To generate His-tagged constructs, DNA fragments of ILK4, ILK5, and GPA1 were PCR-amplified, digested with EcoRI/XhoI, and cloned into the *pET21a* (C terminally His-tagged) or *pET28a* (N terminally His-tagged) vector. Site-directed mutagenesis was performed following the manufacturer’s protocol (Invitrogen, Platinum SuperFi DNA polymerase) to generate ILK4 ^quadruple A^ mutation (Thr326Ala, Ser330Ala, Ser331Ala, and Tyr334Ala) and CBL9^T196A^. To obtain transgenic plants, each construct was introduced into *Agrobacterium tumefaciens* strain *GV3101*, and Arabidopsis plants were transformed using the “floral-dip” method (37).

### Affinity Chromatography for Purification of GST or His-Tagged Proteins

The plasmid was transformed into *Rosetta* (*DE3*) competent cells (Novagen). GST or His-tagged protein was induced using 0.2 mM IPTG. After incubation at 25 °C for 3 h, cells were harvested by centrifugation at 5000*g* for 10 min and the supernatant was discarded. The harvested cells were immediately frozen using liquid nitrogen and thawed on ice. Cells were homogenized in a buffer containing 50 mM Tris–HCl (pH 8.0), 150 mM NaCl, 0.2 mM PMSF, 1 mM DTT, 1 mM EDTA, 0.25 mg/L lysozyme, 1% (v/v) Triton X-100, and protease inhibitor (Thermo Fisher Scientific Protease Inhibitor Mini Tablets EDTA-Free, PIA32955) for the GST-tagged protein. For the His-tagged protein, cells were homogenized in a buffer containing 50 mM Tris–HCl (pH 8.0), 300 mM NaCl, 0.2 mM PMSF, 1 mM DTT, 0.25 mg/L lysozyme, 1% (v/v) Triton X-100, and protease inhibitor (Thermo Fisher Scientific Protease Inhibitor Mini Tablets EDTA-Free, PIA32955). The resuspended total proteins were sonicated twice for 4 min at 4 °C and centrifuged at 12,000*g* for 25 min. The supernatant was transferred to new tubes. GST and His-tagged protein were purified using Glutathione Resin (GenScript #L00206) or TALON metal affinity resin (Takara #635502) following the manufacturer’s protocol.

### Prediction and Selection of the 8000 KiC Library v2.0 Peptides

Over 8000 synthetic peptides were derived from five sources through bioinformatic analysis primarily of experimentally detected Arabidopsis protein phosphorylation events (Fig. 2 and Supplemental Table S2). In all cases, peptides were designed as 20-mers, with phosphosites centered between peptide residues five and 15. Source 1 (phosphopeptides conserved across species): To identify conserved phosphopeptides across species, we conducted a multiple sequence alignment (MSA) with orthologous proteins from Arabidopsis, Medicago, soybean, rice, maize, and lotus, and with evidence of phosphorylation in any of the species in phosphoproteomics data from UniProt or the plant protein phosphorylation database (38).

Phosphosites present in the MSA from two or more species were considered to be conserved independent of the phosphorylated amino acid at a particular phosphosite. This resulted in the following counts of peptides with conserved phosphosites with Arabidopsis in our dataset: *Medicago* (660), *Glycine max* (1105), *Oryza sativa* (536), *Zea Mays* (531), and Lotus (11). After combining these and removing duplicate peptides, a total of 2360 unique peptides resulted. These were further reduced by removing peptides with >60% sequence identity to another peptide in the collection, resulting in 1441 peptides conserved across the angiosperms in our dataset. Source 2 (potentially conserved phosphopeptides): We evaluated the MSA of the proteins containing phosphorylation sites from Arabidopsis, rice, and soybean (31,805 in total) for sequence conservation around the phosphosite with the requirement for potential for phosphorylation (*i.e.*, an S, T, or Y residue) at the same site in orthologs in the MSA. For each orthologous group in the MSA, denoted as *M* of length *L* over *N* (*N* ≥ 3) orthologous sequences. *M*_*C*_ denotes the *C*th column of the alignment, and *M*_*Ci*_ denotes the symbol in column *C* of sequence *i*. *M*_*Ci*_ ∈ *AA*, where *AA* is the 21-element set of amino acids plus the gap (“-”) symbol. *PM*_*Ci*_ denotes the residue conservation score in the sequence around, but excluding each phosphorylation site, according to the Jensen–Shannon divergence (39), which can be defined as below:

$${PM}_{Ci}={\lambda RE}_{p_{C},r}+{\left(1-\lambda \right)RE}_{\left(q,r\right)}$$

where *r* = *λp*_*C*_ + (1 − *λ*)*q* with *q* being the background amino acid distribution, for which we used the total amino acid distribution in the BLOSUM62 alignments. *p*_*C*_ is the column amino acid distribution, which was calculated using the observed frequency of each symbol of *AA* in the column. *λ* is a prior weight. We selected *λ* = 0.5, due to good empirical performance. Relative entropy or the Kullback–Leibler divergence was used to compare probability distributions. The relative entropy conservation score for a column is defined as:

$${RE}_{p_{C},q}=\sum_{\alpha \in AA}p_{C}\left(\alpha \right)\log\frac{p_{C}\left(\alpha \right)}{q\left(\alpha \right)}$$

Following Capra *et al*., we used a sliding window with a length of 11 aa (±5 aa around a phosphorylation site) to evaluate the conservation around each phosphosite (40). The conservation value of the short peptide is defined as:

$${CS}_{p}=\frac{1}{n}\sum_{i=1}^{n}{PM}_{Ci}$$

where *n* is the length of the peptide. This process selected 6604 potentially conserved phosphopeptides for source 2. Source 3: Predicted phosphorylation sites catalyzed by P2K1 and P2K2. We used the 405 peptides that bind P2K1 and P2K2 to build sequence profiles to train HMMER and MusiteDeep, as recently described (41, 42, 43). The trained models were searched against the whole Arabidopsis proteome to predict new candidates for P2K1 and P2K2. The predicted peptides were expanded or trimmed to 20 aa. These candidates (269 in total) form the list of source 3.

Source 4: phosphorylation sites in rice cell wall and development-related genes. We generated 111 peptides based on 240 known phosphorylation sites in the cell wall and development-related genes curated using the trim and centering reformatting methods. Redundant peptides with ≥60% sequence identity to other lists were removed. Source 5 (Root expression): To identify peptides specifically expressed in roots, we obtained root-related genes in Arabidopsis from the TAIR database (1140 genes) and the literature (1189 genes) (2). In total, we obtained 2199 root genes after removing the duplicated entries from the 2339 peptides (1140 + 1189). We then identified all the peptides with phosphorylation sites from these genes. After removing duplicate peptides by using the same 60% identity criteria, this resulted in a list of 1065 peptides. Combining the five sources resulted in 9490 peptides (1441 + 6604 + 269 + 111 + 1065). Several peptides were identified in multiple sources, as depicted in Venn diagram Figure 3 and detailed in Supplemental Table S2. After deduplication, which involves removing completely identical peptides, the unique peptide count stood at 8,000, meeting our target for the design.

### KiC Assay

The KiC assay using the 2k peptide library was previously described (8, 9, 33). To conduct the KiC assay using the 8k peptide library, each 1000-peptide pool was resuspended in dimethyl sulfoxide to an approximate 50 mM final concentration. Next, 10 μl stock aliquots were created and whenever necessary, they were 10× diluted into a working solution of approximately 5 mM. We initially analyzed each 1000-peptide pool without any kinase exposure and investigated whether all peptides could be identified within the library. To ensure the maximum identification number at the mass spectrometry level (DDA acquisition mode), injections of each 1000-peptide pool were made individually. Generally, at least 95% of the synthetic peptides in their neat form could be detected with most charge states being 2^+^ and 3^+^, going up to 6^+^. Subsequently, each set of 1000 peptides was separately tested against the active P2K1 kinase domain, resulting in a total of eight screenings to analyze all 8k peptides for each experimental condition. The purified recombinant GST fused to the P2K1 kinase domain (GST-P2K1-CD) was incubated with the peptide library in the presence or absence of ATP. A kinase-dead version of the P2K1 protein, GST-P2K1^D572N^-CD, was used as a negative control. Thus, each P2K1-CD, WT and dead version, was purified and screened against sets of 1000 peptides in the presence or absence of ATP. Information based on negative control identification results (such as Ascore, PTM ion intensity, and PSM counts) was used to establish efficient filtration criteria for phosphopeptide candidate identification. Results for each screening condition are based on a single biological replicate. To attain optimal enzyme activity, a final 40 μl reaction mixture for the KiC assay was prepared using a kinase buffer composed of 20 mM Tris–HCl (pH 7.5), 10 mM MgCl_2_, 5 mM EGTA, 100 mM NaCl, and 1 mM DTT, with 2 mM ATP only introduced in the "+ATP" samples. Purified P2K1 was quantified using a Bradford assay with Bovine Gamma Globulin as a reference standard. The KiC assay reaction employed a 1:1 enzyme-to-substrate ratio, with 14.72 μg of each added, and the reaction was incubated for 1 h and 30 min at 30 °C with gentle shaking at 300 rpm. Subsequently, the reaction was halted by adding an equivalent volume (40 μl) of a solution containing 1% (v/v) formic acid in 99% (v/v) acetonitrile, and the samples were stored at −20 °C before LC-MS/MS analysis.

### Mass Spectrometry Data Acquisition (Ultrahigh-performance Liquid Chromatography-MS/MS)

Liquid chromatographic separation of peptides was performed using an ultrahigh-performance liquid chromatography EvoSep system with reverse-phase liquid chromatography over a 31-min gradient at a flow rate of 100 nl/min, separating peptides on a C18 analytical column (PepSep C18 Bruker Daltonics, 15 cm × 75 μm, 1.9 μm particle size). Mass spectra were acquired concurrently with chromatographic separation using a DDA method on a TimsTOF Pro 2 (Bruker Daltonics) instrument operating in positive-ion mode with a data-dependent PASEF mode in an *m/z* range of 100 to 1700. PASEF and TIMS were activated. Each cycle, lasting 1.17 s, involved the acquisition of one mass spectrum frame and 10 PASEF frames. The target mass spectrum intensity was set at 10,000 with a minimum threshold of 2500 and collision energies ranging from 20 to 59 eV, using a charge-state-based rolling collision energy table. An active exclusion/reconsider precursor method with release after 0.4 min was employed, and a second tandem mass spectrum was obtained if the precursor exhibited a 4-fold increase in signal strength in subsequent scans (within a mass width error of 0.015 *m/z*).

### Peptide Identification and Data Processing

The PEAKS Studio 10.0 software (https://www.bioinfor.com/peaks-studio/) was employed for automated *de novo* sequencing of MS/MS spectra, with specified precursor and fragment error tolerance values (20 ppm and 0.2 Da, respectively). Variable modifications included phosphorylation of serine, threonine, and tyrosine, as well as oxidation of Methionine, with a maximum of six variable modifications per peptide permitted. Subsequently, the PEAKS DB module within PEAKS Studio was used to identify PSMs from an existing protein database, employing a FASTA file containing 8000 peptide entries as the reference database. The "decoy fusion" target-decoy method integrated into PEAKS was utilized to estimate a 1% false discovery rate for the PEAKS DB results, establishing a confidence threshold for PSMs. In assessing the reliability of phosphorylation site localization, an Ascore was employed, which computes an ambiguity score as −10 × log10 P, where the *p* value signifies the probability of a chance peptide match, making a higher Ascore indicative of greater confidence in the localization. Once phosphopeptide candidates were identified, we ensured high confidence by applying specific filtration criteria, including Ascore ≥20, a minimum PTM ion intensity of ≥5%, at least 2 PSM counts, and a manual examination of the spectrum for clear fragment ions associated with a phosphorylated amino acid. All phosphopeptide candidates were later ranked based on stoichiometry [(phosphopeptide spectrum count/total peptide spectrum count) × 100] and can be assessed in Supplemental Table S3.

### Postidentification Analysis on Phosphopeptide Candidates

Cellular localization information was obtained from the SUBA5 database (http://suba.plantenergy.uwa.edu.au), which combine both computationally predicted and experimentally validated subcellular localization data for numerous Arabidopsis proteins, with a confidence score assigned to each distinct subcellular compartment or region (44). Experimentally determined localizations, derived from methods like mass spectrometry or fluorescent protein–based data, were given higher weight (five times more) than in silico predictions. The final subcellular localization for each gene was determined based on the consensus subcellular location. Functional categorization based on Gene Ontology (GO) Biological Process and GO Molecular Function was conducted for the 8k-peptide library and phosphopeptide candidates, with information retrieved from the TAIR website (45).

### *In Vivo* Phosphoproteomics and Phosphopeptide Candidate Overlap Verification

Three different Arabidopsis genotypes, all in the Col-0 background, were used to perform the phosphoproteomics experiment: WT Col-0, P2K1 mutant (*p2k1-3*), and *P2K1*-overexpression line, (*35S::P2K1/Col-0; OXP2K1*). For phosphoproteomics, the Arabidopsis seedlings were grown on half-strength Murashige and Skoog plates for 10 days, and then treated with 400 μM ATP or mock-treated (buffer only) for 15 min. Crude membrane proteins were isolated as previously described (46). Briefly, seedlings were ground to a fine powder in liquid nitrogen and resuspended in ice-cold homogenization buffer (H buffer, 50 mM Hepes pH 7.5, 250 mM sucrose, 5% (v/v) glycerol, 10 mM EDTA pH 8.0, 0.5% (w/v) polyvinyl pyrrolidone, proteinase, and phosphatase inhibitors) in 50 ml tubes. The samples were centrifugated at 10,000*g* at 4 °C for 10 min, followed by an additional centrifugation at the same speed for 20 min, after which the supernatants were filtered through Miracloth. The filtered supernatants were further centrifugated at 121,000*g*, 4 °C for 4 h to obtain crude membrane pellets. The crude membrane pellets were used for subsequent phosphopeptide enrichment and mass spectrometry analysis.

Fe^3+^-NTA-agarose beads were freshly prepared using the nickel-nitrilotriacetic acid superflow agarose beads (QIAGEN) for the enrichment of phosphopeptide. Tryptic peptides (200 μg) were reconstituted to 0.5 μg/μl in immobilized metal affinity chromatography binding/wash buffer (80% acetonitrile, 0.1% TFA) and incubated with 10 μl of the Fe^3+^-NTA-agarose beads for 30 min at RT with end-over-end turning. After incubation, the beads were washed two times, each with 50 μl of wash buffer and once with 50 μl of 1% formic acid on the stage tip packed with two discs of Empore C18 material (Empore Octadecyl C18, 47 mm; Supleco). Phosphopeptides were eluted from the beads on the C18 stage tip using three 70 μl aliquots of elution buffer (500 mM potassium phosphate buffer). Phosphopeptides were eluted from the C18 stage tips with 50% acetonitrile and 0.1% formic acid. Samples were dried using a Speed-Vac and later reconstituted with 12 μl of 3% acetonitrile, 0.1% formic acid, and 0.01% n-dodecyl β-D-maltoside for LC-MS/MS analysis.

The phosphoproteomics data were collected on a nanoflow liquid chromatography (Dionex Ultimate 3000, Thermo) coupled to a Q-Exactive HF-X mass spectrometer (Thermo) on a C18 75 μm dimension of 30 cm analytical column. The mobile phases were 0.1% formic acid in water for A and 0.1% formic acid in acetonitrile for B. The gradient was 1% to 75% B over 110 min.

Mass spectrometry data acquisition was a data-dependent method of the top 12 precursors with dynamic exclusion of 45 s. Mass spectrum resolution setting was 60,000 for MS1 and 45,000 for MS2. The raw files were searched using MaxQuant 2.0.1.0 (https://www.maxquant.org/) including phosphorylation modifications on STY and oxidation on M. False discovery rate was set to 1%. All data files were uploaded to massive.ucsd.edu with accession MSV000095888.

An alignment search of the 177 phosphopeptide candidates against *in vivo* phosphoproteomics data determined if the detected peptides matched any previously identified phosphorylated peptides. To compare and analyze the peptide sequences, we employed a custom computational approach using the Python programming language and the Biopython library (47). The primary objective was to align sequences derived from experimental data, specifically focusing on phosphorylation sites, which are critical for understanding protein function and regulation.

For sequence alignment, we utilized the global alignment algorithm provided by Biopython’s pairwise2 module. The alignment settings were optimized to maximize similarity, with a scoring system that rewards matches and penalizes mismatches and gaps. The parameters used in the alignment were as follows: a match score of 2 for characters that were the same, a mismatch penalty of −1 for nonmatching characters, a gap opening penalty of −0.5, and a gap extension penalty of −0.1. We also specified the one_alignment_only parameter as true, ensuring that only the best alignment was returned. This method ensured that the most biologically relevant alignments were identified, highlighting conserved regions and potential functional similarities between peptides. After alignment, the results were sorted in descending order based on their alignment scores. This sorting allowed us to prioritize alignments with the highest degree of similarity, suggesting stronger functional relationship between the analyzed peptides. Lastly, we analyzed intensity values from *in vivo* phosphoproteomics data to compare indirect approaches, such as phosphoproteomic experiments, for identifying candidate proteins. Specifically, we employed two inequality-based strategies to select phosphopeptide candidates: a) intensity values: P2K1 mutant (*p2k1-3*) < WT (Col-0) < *P2K1*-overexpression line, and b) intensity values and fold change: P2K1 mutant (*p2k1-3*) < WT (Col-0) (fold change >1.5) < *P2K1*-overexpression line (fold change >1.5).

### *In Vitro* Kinase Assay

An *in vitro* kinase assay was performed with minor modifications as previously described (14, 33). Briefly, 2 μg of purified GST or GST-tagged protein kinases were incubated with 2 μg His-tagged ILK4, ILK5, GPA1, or GST-CBL9 protein as substrates in a 20 μl reaction buffer containing 20 mM Tris–HCl (pH 7.4), 10 mM MgCl_2_, 100 mM NaCl, 2 mM ATP, and with 0.2 μl radioactive [γ-^32^P] ATP for 1 h at 30 °C. The reactions with radioactive [γ-^32^P] ATP were stopped by adding 5 μl of 5 × SDS loading buffer and incubating in a thermomixer (Eppendorf, 22331) at 100 °C for 5 min. Each reaction was separated by electrophoresis in 12% SDS-PAGE gels, and the gel containing radioactive [γ-^32^P] ATP was autoradiographed using a Typhoon FLA 9000 Phospho-imager (GE Healthcare) for 12 h. The reaction mixtures with radioactive [γ-^32^P] ATP were stained with Coomassie Brilliant Blue and used as loading controls.

### Split-LCI Assay

Full-length complementary DNAs constructs were cloned with split-LUC at the C terminus of the *pCAMBIA1300-GW-nLUC* or *pCAMBIA1300-GW-cLUC* vector driven by *CaMV 35S* promoter (33), respectively. The resulting constructs were transformed into *GV3101* using GenePulser electro-transformation (Bio-Rad). When the absorbance (A) reached 1.0, the cultures were suspended in infiltration buffer (10 mM MES, pH 5.7, 10 mM MgCl_2_, and 150 μM 4′-hydroxy-3′,5′-dimethoxyacetophenone) and incubated for 2 h. *GV3101* carrying the constructs (absorbance A = 0.6) was then infiltrated into 4-week-old *N. benthamiana* leaves. The infiltrated leaves were incubated at 28 °C for 3 days before LUC activity measurement. To quench the fluorescence, 5 mM D-luciferin containing 0.01% (v/v) Triton X-100 was sprayed onto the *N. benthamiana* leaves and placed in the dark for 10 min. The luminescence was monitored and captured using a low-light imaging CCD camera (Photek; Photek, Ltd).

### Coimmunoprecipitation Assay

Agrobacterium *GV3101* harboring the specified constructs in infiltration buffer [10 mM MES (pH 5.7), 10 mM MgCl_2_, 150 μM 4′-hydroxy-3′,5′-dimethoxyacetophenone] was coinfiltrated into 4-week-old *N. benthamiana* leaves. Total protein was extracted from the coinfiltrated *N. benthamiana* leaf tissues using the following protein extraction buffer: 50 mM Tris–HCl (pH 7.5), 150 mM NaCl, 0.2 mM PMSF, 0.5% (v/v) Triton-X 100, and 1× protease inhibitor (Thermo Fisher Scientific; A32955) with gentle agitation at 4 °C for 2 h. The solution was then centrifuged at 20,000*g* for 15 min at 4 °C. The supernatant was transferred to a new e-tube, and 30 μl of monoclonal anti-hemagglutinin (HA) antibody agarose beads (Sigma, A2095, 1 ml) were added, followed by overnight incubation with end-to-end shaking at 4 °C. Subsequently, the beads underwent at least seven washes with a washing buffer containing 50 mM Tris–HCl (pH 7.5), 150 mM NaCl, and 1× protease inhibitor. After washing, the resin was eluted with 25 μl of 1× SDS-PAGE loading buffer, and the eluent was heated in boiling water for 10 min. The proteins were separated using 10% SDS-PAGE and detected by immunoblotting with anti-HA-horseradish peroxidase (Roche, 12013819001; dilution, 1:1000) and anti-Myc (Sigma-Aldrich, SAB4700447; dilution, 1:2000) antibodies.

### Protoplast Isolation and BiFC Assay

YFP or split-YFP protein fusion plasmids (*pAM-PAT-GW-YFP* or *pAM-PAT-YFP-GW* for YFP and *pAM-PAT-GW-nYFP* and *pAM-PAT-GW-cYFP* for split-YFP) were introduced into Arabidopsis protoplasts by PEG-mediated transformation, as previously described (33, 48). The transformed protoplasts were incubated in a growth chamber at 21 °C for 24 h in the dark. YFP fluorescence was observed using a Leica DM 5500B compound microscope with a Leica DFC290 color digital camera 24 h after transformation. A counterstain, 5 μM FM4-64 (Invitrogen, T3166), was used for the plasma membrane. For YFP transgenic plants, YFP constructs driven by the *CaMV 35S* promoter were transformed into *A. tumefaciens GV3101* (*pMP90RK*), and Arabidopsis plants were transformed using the "floral-dip" method (37). Confocal images were generated using a Leica TCS SP8 STED laser confocal microscope attached to a vertical microscope (Leica MP Color Digital Camera) equipped with various fluorescein filters. The YFP signal was excited at a wavelength of 514 nm under a laser-scanning confocal microscope with an argon ion laser system.

### *In Vivo* Phosphorylation Assay

*In vivo* phosphorylation assays were performed as previously described (33). Briefly, 100 μg of pUGW-ILK4-HA constructs were introduced into *Arabidopsis* protoplasts extracted from Col-0, *p2k1-3*, or *P2K1*-overexpressing plants (*OXP2K1*) *via* PEG-mediated transformation, using leaf tissues from four-week-old plants. Protoplasts were incubated at 21 °C in the dark for 48 h with 2 ml W5 solution (2 mM MES pH 5.7, 154 mM NaCl, 125 mM CaCl_2_, and 5 mM KCl). After adding 250 μM ATPγS (poorly hydrolyzed ATP analog) and incubating for 1 h, protoplasts were harvested by centrifugation at 50*g* for 4 min. The supernatant was removed, and 250 μl of protein extraction buffer [50 mM Tris–HCl (pH 7.5), 150 mM NaCl, 0.2 mM PMSF, 0.5% Triton-X 100, and 1× protease inhibitor] was added, followed by 2 h incubation at 4 °C. Immunoprecipitations were performed as described above, and proteins were separated by SDS-PAGE, followed by immunoblotting with anti-HA-horseradish peroxidase (Roche, 12013819001; 1:2000) and anti-phospho-Ser/Thr (BD Transduction laboratories, 612548, 1:8000) antibodies.

### MAPK Phosphorylation and Immunoblot Assay

Total protein was extracted from leaf discs of 4-week-old plants at the indicated time points following treatment with 250 μM ATP *via* homogenization. Protein extraction was performed using a buffer containing 50 mM Tris–HCl (pH 7.5), 150 mM NaCl, 10 mM MgCl_2_, 1 mM EDTA, 1 mM DTT, 0.2 mM PMSF (Sigma, 93482), 10% (v/v) glycerol, 0.5% (v/v) Triton-X 100, and 1× protease inhibitor (Sigma-Aldrich, PIA32955). The samples were gently agitated at 4 °C for 2 h. After extraction, the samples were centrifuged at 20,000*g* for 15 min at 4 °C. The supernatant was transferred to an e-tube and centrifuged again at 20,000*g* for 10 min at 4 °C to pellet any remaining leaf debris. The extracted proteins were mixed with 5× Laemmli loading buffer containing 10% (w/v) SDS, 50% (v/v) glycerol, 0.01% (w/v) bromophenol blue, 10% (v/v) β-mercaptoethanol, and 0.3 M Tris–HCl (pH 6.8), and heated in boiling water for 5 min. Proteins were separated by 10 or 12% SDS-PAGE and detected by immunoblotting using an anti-phospho-p44/p42 MAPK antibody (Cell Signaling, 4370L; dilution 1:2000).

### GUS Assay

Wounded leaf tissues were submerged in a histochemical staining solution containing 1 mM 5-bromo-4-chloro-3-indolyl β-glucuronic acid, 100 mM sodium phosphate (pH 7.0), 0.1 mM EDTA, 0.5 mM ferricyanide, 0.5 mM ferrocyanide, and 0.1% (v/v) Triton-X 100. After a vacuum incubation for 10 min, the seedlings were further incubated at 37 °C for 6 to 12 h, depending on the staining status. For the wounding treatment, rosette leaves were wounded using hemostat forceps. Chlorophyll was removed from the plant tissues by immersion in 70% ethanol and then repeatedly washing with 70% ethanol until the tissue became clear. Stained tissues were observed using a Fisher Stereo-master microscope (FW02-18B-1750), and digital images were captured with an AmScope digital camera (MU1000).

## Results

### Discovery of P2K1 Kinase Substrates Using the 2k Peptide Library

**Fig. 1:**
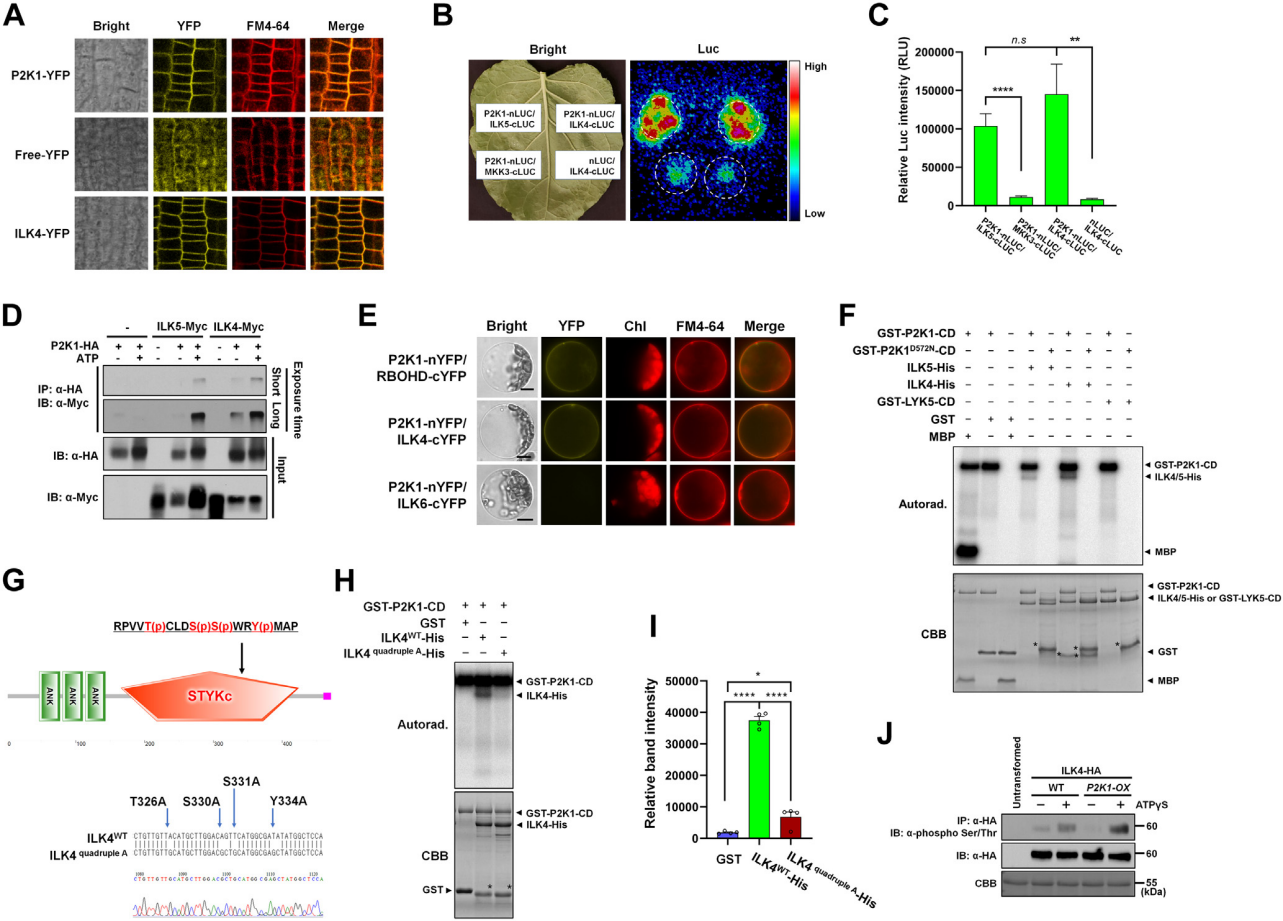
**ILK4, which was identified through the KiC assay with the 2k peptide library, interacts with and is phosphorylated by P2K1 kinase domain.***A*, subcellular localization of ILK4 in transgenic plants expressing a *35S:ILK4*-*YFP* construct. Fluorescent confocal images displaying the subcellular distribution of ILK4-YFP protein were detected from primary root tissue of seven-day-old seedlings. Plasma membrane was counter-stained by incubation for 1 min in a FM4-64 solution (5 μM). P2K1-YFP and free-YFP were used as controls. *B*, the split-luciferase assay provides evidence that P2K1 interacts with ILK4. The emitted luminescence from the leaves was captured using a low-light imaging CCD camera (Photek; Photek, Ltd). *Dotted circles* indicate the regions of interest in *N. benthamiana* leaves corresponding to the infiltrated areas. ILK5-cLUC and MKK3-cLUC proteins served as positive and negative controls, respectively. *C*, the signal intensities of the P2K1–ILK4 interaction were quantified. Captured images were analyzed for luciferase signal intensities using C-vision/Im32 software, and further data analysis was performed with GraphPad Prism (version 8). The results are presented as the mean ± SEM of eight biological replicates. Statistical significance was determined by unpaired two-tailed Student’s *t* test, with significance levels denoted as follows: ∗∗∗∗*p* < 0.0001, ∗∗∗*p* < 0.001, ∗∗*p* < 0.01, and ∗*p* < 0.05. *D*, coimmunoprecipitation (Co-IP) was carried out to investigate the interaction between P2K1 and ILK4 proteins in *N. benthamiana* leaves. Total protein was utilized for the Co-IP assay with anti-HA and anti-Myc antibodies. ILK5-Myc was employed as a positive control. *E*, a BiFC assay was conducted in Arabidopsis protoplasts. FM4-64 was employed as a plasma membrane marker, and the chlorophyll autofluorescence signal (Chl) was also observed. The merged image (Merge) represents the overlapping fluorescence signals from YFP and FM4-64. RBOHD-cYFP and ILK6-cYFP were used as positive and negative controls, respectively. Scale bars represent 10 μm. *F*, GST-P2K1-CD directly phosphorylates ILK4-His, whereas GST-LYK5-CD is not phosphorylated *in vitro*. In an *in vitro* kinase assay, bacterial recombinant ILK4-His protein was incubated with GST-P2K1-CD, GST-P2K1^D572N^-CD (a kinase-dead version), or GST. *G*, a schematic diagram of ILK4 depicts its structural features, including the ankyrin repeat (ANK) and serine-threonine or tyrosine kinase (S-T/Y_kinase) domains. The identified phosphopeptide RPVVT(p)CLDS(p)S(p)WRY(p)MAP is situated within the kinase domain. *H*, mutation of ILK4 at the residues T326, S330, S331, and Y334 resulted in diminished phosphorylation by P2K1 *in vitro*. The *in vitro* kinase assay involved the incubation of purified GST or GST-P2K1-CD recombinant proteins with ILK4^WT^-His or ILK4 ^quadruple A^-His. *I*, quantification of phosphorylated ILK4^WT^ and ILK4^quadruple A^ proteins was performed. Phosphorylation signal intensities were measured and analyzed using ImageJ and the GraphPad Prism 8 software. The data is presented as mean ± SEM (n = 4) based on measurements from four independent experiments. Significance levels are represented as ∗∗*p* < 0.01, with the *p* value indicating the significance concerning the band intensity of ILK4^WT^-His, determined through unpaired two-tailed Student’s *t* test. In panels (*E* and *G*), the presence of autophosphorylation and transphosphorylation events was identified by the incorporation of [γ-^32^P] ATP. *Asterisk* (∗) represents the nonspecific protein. Myelin basic protein (MBP) was used as a universal substrate, while GST-LYK5-CD was used as a negative control. Protein loading was visualized through Coomassie Brilliant Blue (CBB) staining. *J*, ILK4 phosphorylation is significantly increased in *P2K1*-overexpressing plants. ILK4-HA protein was expressed in WT and *P2K1*-overexpressing plants protoplasts with/without 250 μM ATP treatment and then subjected to immunoprecipitation and IB using anti-HA and anti-phospho-Ser/Thr antibodies. Protein loading was visualized by CBB staining. All of the above experiments were repeated two times (biological replicates) with similar results. BiFC, bimolecular fluorescence complementation; CD, cytosolic domain; GST, glutathione-*S*-transferase; IB, immunoblotting; ILK, integrin-linked kinase; KiC, kinase client; RBOHD, respiratory burst oxidase homolog D.

To identify substrate proteins of the P2K1 kinase, we employed a mass spectrometry–based *in vitro* phosphorylation method called the KiC assay (7, 9). The KiC assay was performed with purified recombinant N terminally fused GST-P2K1 cytosolic domain (GST-P2K1-CD), with confirmed kinase activity (Supplemental Fig. S1). In these initial assays, we used a synthetic peptide library containing 2k experimentally detected *in vivo* phosphorylation sites (14, 33, 34). The 2k peptide library was incubated with recombinant GST-P2K1-CD in the presence of ATP. Subsequently, tandem mass spectrometry identified phosphorylated peptides, including the specific phosphorylation residue on each peptide. This experiment identified phosphorylated peptides based on phosphoRS scores and phosphoRS site probabilities (9, 14, 49). Previous publications described validation of four P2K1 kinase substrates identified from this screening, including RBOHD, protein S-acyltransferase 5/9 (PAT5/9), and ILK5 (14, 33, 34). The peptide RPVVT(p)CLDS(p)S(p)WRY(p)MAP from ILK4 was also phosphorylated by the P2K1 kinase domain in the 2k KiC assay (Table 1). ILK4 is the closest homologous protein to ILK5. It is composed of an ANK domain at the N terminus and an S/T/Y kinase domain at the C terminus (Supplemental Fig. S2*A*). Similar to P2K1 and ILK5 (33), ILK4 is also upregulated in response to wounding stress (Supplemental Fig. S2, *B* and *C*). Furthermore, based on SUBA, ILK4 is predicted to be localized to the plasma membrane similarly, to P2K1 (24). ILK4 tagged with YFP at the C- or N-terminal YFP expressed in Arabidopsis plants and protoplasts confirmed that the majority of ILK4 colocalized with the FM4-64, a plasma membrane marker (Fig. 1*A* and Supplemental Fig. S2*D*).

**Table 1:** Putative kinase clients identified by KiC assay using a synthetic peptide library consisting of 2k peptides as substrates for P2K1 kinase

Accession	Phospho peptide	pRS^a^	pRS site probability^b^	Protein annotation^c^	Interaction	Methods	References
At1g01550	**S**MGA**S**TLQATSPKKAAG	143	S(1): 100.0; S(5): 0.0	Bypass 1	TBD		
At3g48760	GAP**SSS**GGVSGGDELIRTY	115	S(4): 7.9; S(5): 7.9; S(6): 84.3	DHHC-type zinc finger family protein (PAT5)	Y	BiFC, Co-IP, GST pull-down assay	Chen et al., 2021
At1g53050	RQ**T**QPL**T**SRVVTLWY	87	T(3): 99.7; T(7): 0.2	Protein kinase superfamily protein	N	LCI assay	
At2g38280	VRPI**S**PK**S**PVASASAF	79	S(5): 98.6; S(8): 1.4	Embryonic factor1	N	LCI assay	
At5g15160	NLNKEADDL**S**DRL**T**QLL	72	S(10): 91.8; T(14): 8.2	BHLH134	Y	BiFC, LCI, GST pull-down assay	
At5g43310	E**S**IA**T**GIRLTRSISPLPLS	62	S(2): 99.9; T(5): 0.1	COP1-interacting protein-related	N	LCI assay	
At3g04470	AAEEEF**ST**PP**S**SPVFHDAK	60	S(7): 89.8; T(8): 10.0; S(11): 0.2	Ankyrin repeat family protein	TBD		
At5g47910	GILRGAN**S**D**T**NSDTESI	53	S(8): 99.8; T(10): 0.2	AtRBOHD	Y	BiFC, Co-IP, GST pull-down assay	Chen et al., 2017 (14)
At5g55900	FLAE**S**I**S**R**S**GSFESGSLR	53	S(5): 79.8; S(7): 9.4; S(9): 9.4	Sucrase/ferredoxin-like family protein	TBD		
At5g35750	LADEVKEPL**T**IEDAVL	48	T(10): 100.0	Histidine kinase 2	Weak or N	LCI assay	
At1g21580	LSPEND**SS**RGRPMGL	47	S(7): 4.2; S(8): 95.7	Zinc finger C-x8-C-x5-C-x3-H type family	TBD		
At4g13420	WGKL**Y**RPD**S**FII	46	Y(5): 10.5; S(9): 89.5	ATHAK5	N	LCI assay	
At4g18950	VKKLDDEVL**S**	43	S(10): 100.0	Integrin-linked protein kinase family (ILK5)	Y	BiFC, Co-IP, LCI assay	Kim et al., 2023
At5g43310	E**S**IA**T**GIRL**T**RSISPLPL	41	S(2): 98.0; T(5): 1.9; T(10): 0.1	COP1-interacting protein-related	N	LCI assay	
At5g50020	**T**V**S**SDGRQTPTVQIPR	33	T(1): 77.4; S(3): 22.1	DHHC-type zinc finger family protein (PAT9)	Y	BiFC, Co-IP, GST pull-down assay	Chen et al., 2021 (34)
At5g09390	DAWLD**S**IEKNPM**Y**MGR**S**A	31	S(6): 80.7; Y(13): 9.6; S(17): 9.6	CD2-binding protein-related	N	GST pull-down assay	Chen et al., 2021 (34)
At3g20350	SPVSHA**S**KAH**T**V**S**PDVNLI	27	S(7): 20.9; T(11): 69.2; S(13): 95.1	unknown protein	TBD		
At1g79280	DPKRAP**S**PGGGSS**T**IV**T**LA	23	S(7): 72.6; T(14): 71.9; T(17): 95.6	Nuclear pore anchor	TBD		
At3g58760	RPVV**T**CLD**SS**WR**Y**MAP	17	T(5): 74.1; S(9): 16.9; S(10): 16.9; Y(13): 92.1	Integrin-linked protein kinase family (ILK4)	Y	BiFC, Co-IP, LCI assay	Current study
At3g28690	**SS**LNLPQASPYR**Y**ARQ	15	S(1): 94.2; S(2): 94.2; S; Y(13): 88.6	Protein kinase superfamily protein	TBD		
At5g08080	G**S**FELPRGQ**SS**REGDVELG	15	S(2): 84.9; S(10): 84.9; S(11): 30.2	Syntaxin of plants 132	TBD		

BiFC, bimolecular fluorescence complementation; Co-IP, coimmunoprecipitation; GST, glutathione-*S*-transferase; KiC, kinase client; LCI, luciferase complementation imaging; RBOHD, respiratory burst oxidase homolog D.

a pRS score: This peptide score is based on the cumulative binomial probability that the observed match is a random event. The value of the pRS score strongly depends on the data scored, scores 15 was considered as potential client.

b pRS site Probabilities: For each phosphorylation site this is an estimation of the probability (0–100%) for the respective site being truly phosphorylated. pRS Site Probabilities above 50% are good evidence that the respective site is truly phosphorylated.

c Protein annotation from TAIR database. TBD, Y, and N represent to be determined, Yes and No, respectively.

### P2K1 Interacts with and Phosphorylates ILK4

We then conducted a split-LCI assay to test whether P2K1 and ILK4 interact. In this assay, we coinfiltrated *N. benthamiana* leaves with *P2K1-nLUC* and *ILK4-cLUC* constructs. The results confirmed the interaction of P2K1 with ILK4 *in planta*. ILK5 and MKK3 were used as a positive and a negative control, respectively (Fig. 1, *B* and *C*).

Consistent with a role in eATP signaling, we demonstrated that the interaction between P2K1 and ILK4 was enhanced upon the addition of ATP. For this, we coexpressed HA-tagged P2K1 with a Myc-tagged ILK4 or ILK5 in *N. benthamiana* leaves in the presence and absence of exogenous ATP, followed by immunoprecipitation using antibodies to the specific epitope tags. P2K1 coimmunoprecipitated with ILK4 or ILK5, and their interaction appeared to be enhanced in the presence of eATP and confirmed using split-LCI assay (Fig. 1*D* and Supplemental Fig. S2*E*). BiFC assays were conducted in Arabidopsis protoplasts demonstrating the interaction of P2K1 with ILK4 at the plasma membrane. Coexpression of P2K1-nYFP with ILK4-cYFP produced a yellow fluorescent signal that colocalized with the plasma membrane marker FM4-64, whereas coexpression of P2K1-nYFP with the control ILK6-cYFP protein did not display a yellow fluorescent signal (Fig. 1*E*). The data are consistent with a physical interaction between P2K1 and ILK4 at the plasma membrane.

To confirm that ILK4 is a client of P2K1’s kinase activity, purified His-tagged recombinant ILK4 (ILK4-His) protein was incubated with either purified GST-tagged P2K1 CD (GST-P2K1-CD) or a kinase-dead version of P2K1 (GST-P2K1^D572N^-CD) in the presence of [γ-^32^P] ATP. The results show that GST-P2K1-CD transphosphorylated ILK4-His, but not GST-LYK5-CD protein (14, 50), which represents a negative control (Fig. 1*F*). Assays performed with the kinase-dead version of P2K1 failed to phosphorylate ILK4-His (Fig. 1*F*). ILK5 and myelin basic protein were used as a specific and universal positive control substrate, respectively. To verify that the radiographic signal was the result of phosphorylation, the addition of lambda protein phosphatase significantly reduced both autophosphorylation and transphosphorylation of P2K1 and ILK4 (Supplemental Fig. S3). The MS/MS results from the KiC assay revealed that the Thr326, Ser330, Ser331, and Tyr334 residues of ILK4 were the specific targets of P2K1 phosphorylation (Fig. 1*G*). To confirm this, site-directed mutagenesis was used to generate an ILK4^quadruple A^ recombinant protein, in which all four of these potentially phosphorylated residues were replaced with alanines. Incubation of ILK4^quadruple A^ recombinant protein with GST-P2K1-CD showed a significant reduction in phosphorylation, compared to the WT protein as expected (Fig. 1, *H* and *I*). To investigate whether ILK4 phosphorylation is dependent on P2K1, ILK4-HA protein was expressed in Col-0 or *P2K1*-overexpressing plants (*OXP2K1*) under ATPγS treatment followed by immunoprecipitation using anti-HA antibody. Immunoblotting was subsequently performed with an anti-phospho-Ser/Thr antibody. The ILK4-HA protein was phosphorylated in the presence of ATPγS, and phosphorylation of the ILK4-HA protein was significantly increased in *P2K1*-overexpressing plants compared to WT plants (Fig. 1*J*).

### Involvement of ILK4 in MAPK Activation

**Fig. 2:**
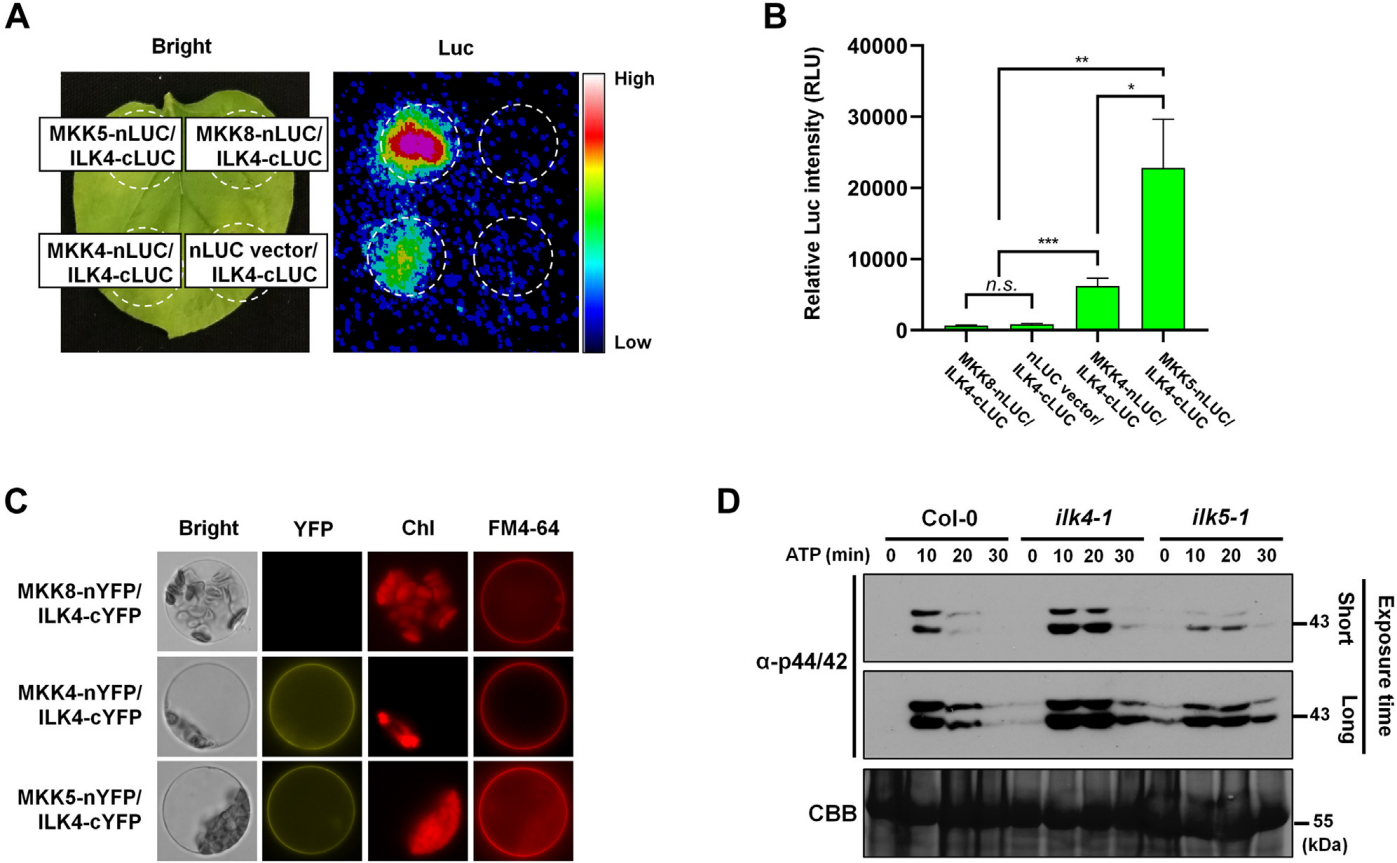
**ILK4 interacts with MKK4 and MKK5.***A*, the interaction between ILK4 and MKK4 or MKK5 were assessed *via* a split-LUC assay. *Dotted circles* indicate the regions of interest corresponding to the infiltrated areas in *N. benthamiana* leaves. MKK8-nLUC/ILK4-cLUC and nLUC vector/ILK4-cLUC proteins were employed as negative controls. *B*, the signal intensities of the ILK4-MKK4 or ILK4-MKK5 interaction were quantified using C-vision/Im32 software and analyze with GraphPad Prism. The results are presented as the mean ± SEM of eight biological replicates. Statistical significance was determined by unpaired two-tailed Student’s *t* test, with significance levels denoted as follows: ∗∗∗∗*p* < 0.0001, ∗∗∗*p* < 0.001, ∗∗*p* < 0.01, and ∗*p* < 0.05. *C*, a BiFC assay was conducted in Arabidopsis protoplasts. FM4-64 was used as a plasma membrane marker, and Chl represents chlorophyll autofluorescence. The negative control was provided by MKK8-nYFP/ILK4-cYFP. Scale bars represent 10 μm. *D*, phosphorylation of MPK3/6 was detected in *ilk4-1* and *ilk5-1* mutants by immunoblotting using anti-phospho 44/42 antibody. ATPγS (250 μM) was added and incubated for the times shown. Total protein was extracted at each time point and immunoblotting (IB) was performed with an anti-phospho 44/42 MAPK antibody. CBB staining of protein was used as a loading control. These experiments were biologically repeated two times with similar results. BiFC, bimolecular fluorescence complementation; CBB, Coomassie Brilliant Blue; ILK, integrin-linked kinase.

ILK5 interacts with MKK4 and MKK5 to activate MAPK cascades in response to the bacterial pathogen *P. syringae* DC3000, thereby regulating plant immune responses (33). ILK4 is classified as a Raf-like MAPKKK and is the closest homolog to ILK5 (51). Accordingly, we investigated the interactions of ILK4 with the 10 Arabidopsis MKKs using the split-LCI assay and found that, interestingly, ILK4 interacts with MKK4 and MKK5, similar to ILK5 (Fig. 2, *A* and *B* and Supplemental Fig. S4). BiFC assays conducted in Arabidopsis protoplasts confirm the interaction of ILK4 with MKK4 or MKK5 at the plasma membrane. Coexpression of MKK4-nYFP or MKK5-nYFP with ILK4-cYFP produced a yellow fluorescent signal that colocalized with the plasma membrane marker FM4-64; whereas, coexpression of ILK4-cYFP with the control MKK8-nYFP protein did not display a yellow fluorescent signal (Fig. 2*C*). The data are consistent with a interaction between ILK4 and MKK4 or MKK5 at the plasma membrane. To detect the phosphorylation of downstream MPK3 and MPK6 in ATP-treated plants, samples were harvested at 10-min intervals following ATP treatment of 4-week-old plants. Total protein was then extracted, and immunoblotting was performed using an anti-phospho-p44/p42 MAPK antibody, with Coomassie Brilliant Blue serving as a loading control. The *ilk5-1* mutant exhibited decreased phosphorylation of MPK3 and MPK6 under ATP treatment compared to Col-0, while the *ilk4-1* mutant showed a slight increase in phosphorylation relative to Col-0 (Fig. 2*D*). This suggests that, unlike ILK5, ILK4 may act as a negative regulator or a finely tuned modulator of excessive MAPK activation within the signaling cascades.

### Design and Construction of the 8k Peptide Library for Expanded KiC Assay

**Fig. 3:**
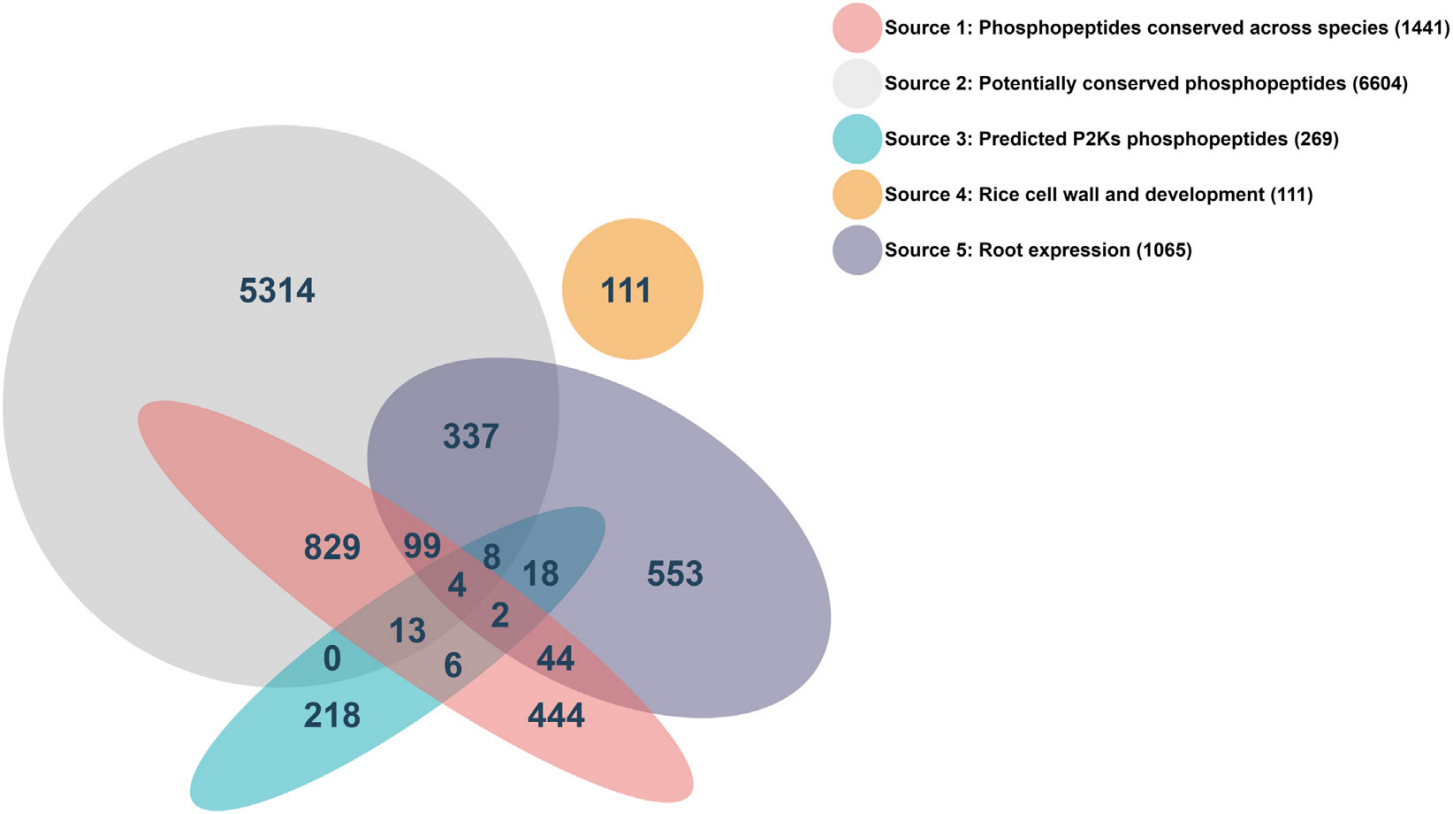
**Venn diagram of 8k peptide library.** 8k peptides are categorized based on bioinformatic predictions and functions. Source 1: phosphopeptides conserved across species (1441): This represents the set of phosphopeptides that are conserved across different species, totaling 1441 unique peptides. Source 2: potentially conserved phosphopeptides (6604): Here, we have a larger set that includes phosphopeptides that are potentially conserved, with a total of 7772 peptides. Source 3: predicted P2Ks phosphopeptides (269): This category includes predicted phosphopeptides that are catalyzed by P2Ks which have 269 peptides. Source 4: rice cell wall and development (111): This subset is specific to rice, focusing on phosphopeptides involved in cell wall composition and developmental processes, with 111 peptides. Source 5: root expression (1065): Representing phosphopeptides found to be expressed in roots, the total count here is 1065 peptides. Overlap areas: The numbers in the overlapping areas of the circles represent phosphopeptides that are common between different sources. For example, the overlap between source 1 and source 2 (*blue and gray circles*) contains 1273 phosphopeptides that are found to be conserved across species and also considered as potentially conserved. The individual numbers inside each circle or overlapping areas correspond to the number of phosphopeptides unique to that specific category or shared between categories, as indicated by the position of the number in the Venn diagram. That information could also be retrieved from Supplemental Table S2.

As demonstrated by the example of ILK4 above and in previous studies, the use of the 2k peptide library allowed us to successfully identify and isolate physiological substrates for the P2K1 protein (14, 33, 34). To extend this success and discover additional substrates, we expanded our library to approximately 8000 (8k) peptides. The new 8k peptide library consists of 20-mers designed primarily based on experimentally observed phosphosites in Arabidopsis, with phosphorylation sites centered between residues 6 to 14 of the peptide. Our selection from among the approximately 32k Arabidopsis 20-mer phosphopeptides represented in public data was based on evidence of conservation or potential for conservation along with a handful of other criteria (Fig. 3 and Supplemental Fig. S5).

We reasoned that phosphorylated residues observed in multiple species might be more likely to represent functional sites with regulatory roles compared with nonconserved sites. MSAs of proteins with phosphorylated residues from six angiosperms revealed only 1441 peptides, when overlapping or largely similar sequences were removed (source 1). This relatively small number might reflect that plant protein phosphorylation beyond Arabidopsis, at least, is still relatively undersampled. To expand this number toward our target, we examined the MSA of Arabidopsis phosphoproteins for phosphosites that have the potential to be phosphorylated in other species based on having an S-, T-, or Y- residue at the same location as the phosphosite in orthologs and sequence similarity for the surrounding amino acid residues. The 10,264 potentially conserved Arabidopsis peptides were than ranked based on conservation score and other criteria relevant to our scientific interests to fit within the goal of 8k peptides, ultimately trimming the list of potentially conserved peptides to 6604 peptides (source 2). Thus, about 83% of the 8k library represents conserved or potentially conserved phosphorylated plant peptides.

We used some additional criteria to prioritize among the potentially conserved phosphopeptides and added other peptides. First, we used bioinformatics analysis to predict peptides that might be substrates for P2K1 and P2K2, selecting 269 of these predicted peptide substrates (source 3). Only a handful of these (33) overlapped with the selected conserved or potentially conserved peptides (Fig. 3). We manually selected 111 phosphorylated peptides from rice related to internode development (source 4). Finally, due to the importance of roots as an organ for purinergic signaling (24, 52), we prioritized 1065 phosphopeptides with gene expression evidence from Arabidopsis roots (source 5). From among all these lists many, but not all, peptides with overlapping sequences (*i.e.*, adjacent phosphosites) or high sequence similarity were removed for a final number of 7990 unique peptides within the 8k peptide library (Supplemental Table S2). Hence, although largely based on Arabidopsis peptides, the 8k library may be enriched for conserved, functionally relevant clients and may also be useful for screening against kinases from other plant species.

GO molecular function and biological process analysis of the protein represented by the 8k peptides revealed the presence of genes with various functions involved in diverse cellular processes, including response to stress and protein catalytic activity (Supplemental Fig. S6, *A* and *B* and Supplemental Table S4). Additionally, SUBA analysis of the subcellular localization of the protein represented by the 8k peptides indicates a diverse distribution across subcellular compartments, especially the nucleus, cytosol, and plasma membrane (Supplemental Fig. S6*C* and Supplemental Table S5).

### Use of the 8k KiC Library to Discover Novel Substrates for P2K1

**Fig. 4:**
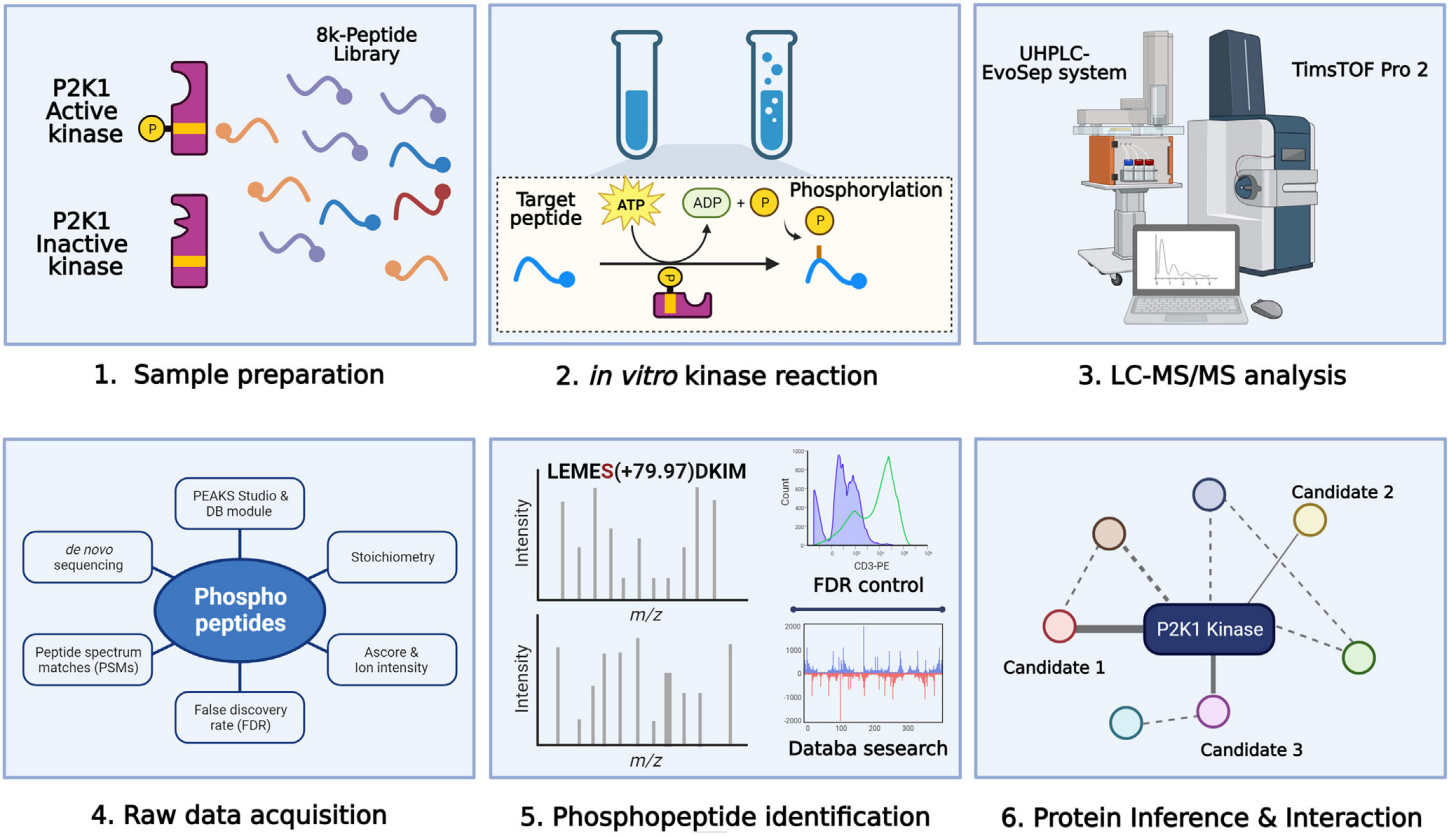
**Schematic of main steps involved in the KiC assay screening of peptide libraries.***1,* sample preparation, which includes the kinase purification and activity test as well as preparation of the 8k synthetic peptide library prior to screening. *2, in vitro* kinase reaction in which optimal conditions are provided for phosphorylation events to occur. *3,* sample analysis on an LC-MS/MS. *4,* raw data acquisition. *5,* phosphopeptide identification achieved by database searches. Thorough filtration is conducted to enable high-confidence phosphorylation and site determination identifications using PEAKS software (https://www.bioinfor.com/peaks-studio/). *6,* functional characterization of phosphopeptide candidate identifications. This figure was created using BioRender (https://biorender.com/). KiC, kinase client.

**Fig. 5:**
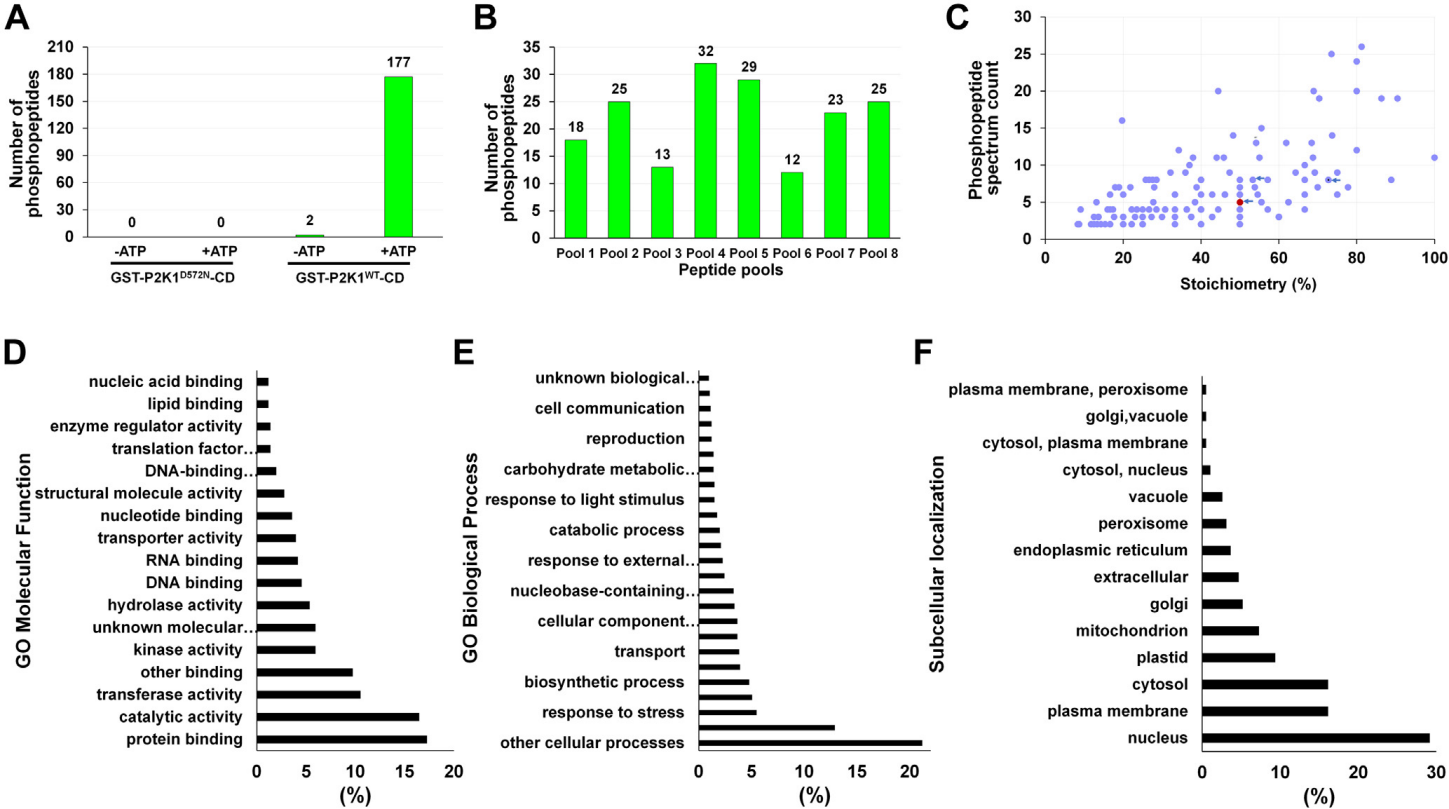
**Screening results of P2K1 enzyme against an 8k peptide library pool.***A*, number of phosphopeptide candidates across different experimental conditions that showed an Ascore ≥20, PTM minimum ion intensity ≥5%, PSM counts ≥2, and passed a manual spectrum check. *B*, phosphopeptide identification distribution according to P2K1 screening results in each 1000-peptide pool for the WT + ATP screening condition. *C*, phosphopeptide ranking classification of P2K1 screening results based on stoichiometry and phosphopeptide spectrum count (stoichiometry = (phosphopeptide spectrum count)/(total peptide spectrum count) × 100). The *arrows* represent peptides CBL9 (*red dot*), ATL2, and LRR-RK. *D* and *E*, functional categorization by annotation for GO molecular function and GO Biological Process among the novel 177 phosphopeptide clients. *F*, analysis of subcellular localization of the 177 phosphopeptide clients using SUBA5 software. CBL9, calcineurin B–like protein 9; GO, Gene Ontology; LRR-RK, leucine-rich repeat receptor-like kinase protein; PSM, peptide spectrum match; PTM, posttranslational modification.

To identify additional substrates of the P2K1 kinase, we performed the KiC assay with the new 8k peptide library. The purified, recombinant GST-P2K1-CD or GST-P2K1^D572N^-CD from *BL21 DE3* (YopH phosphatase) were incubated with ATP and the 8k peptide library divided into eight 1k-peptide pools, and the products were analyzed using LC-MS/MS (Fig. 4). After filtration, the analysis identified a total of 177 phosphopeptides phosphorylated (185 phosphorylated residues) by P2K1 (Fig. 5*A* and Supplemental Table S3). As expected, a background of only two phosphopeptides were detected in the KiC assay reaction when ATP was absent from GST-P2K1-CD (Fig. 5*A*). The kinase-dead version of P2K1 (GST-P2K1^D572N^-CD) also produced no phosphopeptides, with or without ATP (Fig. 5*A*). The 177 phosphorylated peptides were distributed among the eight KiC assay pools (Fig. 5*B*). We further classified the phosphopeptides based on stoichiometry and phosphopeptide spectrum count (Fig. 5*C*). Stoichiometry assesses the ratio of phosphorylated spectra matches for a specific phosphopeptide relative to the overall number of PSMs, encompassing both phosphorylated and nonphosphorylated spectra. Phosphopeptide candidates identified with a high stoichiometry suggest the kinase has a low K_m_ for the peptide substrate (Fig. 5*C*). GO molecular function and biological process analysis of the candidate peptides revealed that peptides associated with genes possessing catalytic activity, protein binding, transferase activity, response to stress, and various cellular processes were frequently phosphorylated. Peptides related to the functions of proteins involved in the regulation of protein activity, binding, and transport were also found to be phosphorylated (Fig. 5, *D* and *E* and Supplemental Table S4). SUBA analysis of the subcellular localization of the 177 phosphorylated peptides' proteins revealed that they were distributed in various cellular compartments, including the cytosol, plasma membrane and nucleus (Fig. 5*F* and Supplemental Table S5). In addition, analysis of the shared sequences in phosphorylated peptides revealed a motif of shared residues; specifically, hydrophobic amino acids such as Trp (W) or Phe (F) were prominently distributed around phosphorylated Ser and/or Thr. (Supplemental Fig. S7).

### CBL9 and GPA1, Newly Identified as Putative P2K1 Substrates Using the 8k KiC Peptide Library

**Fig. 6:**
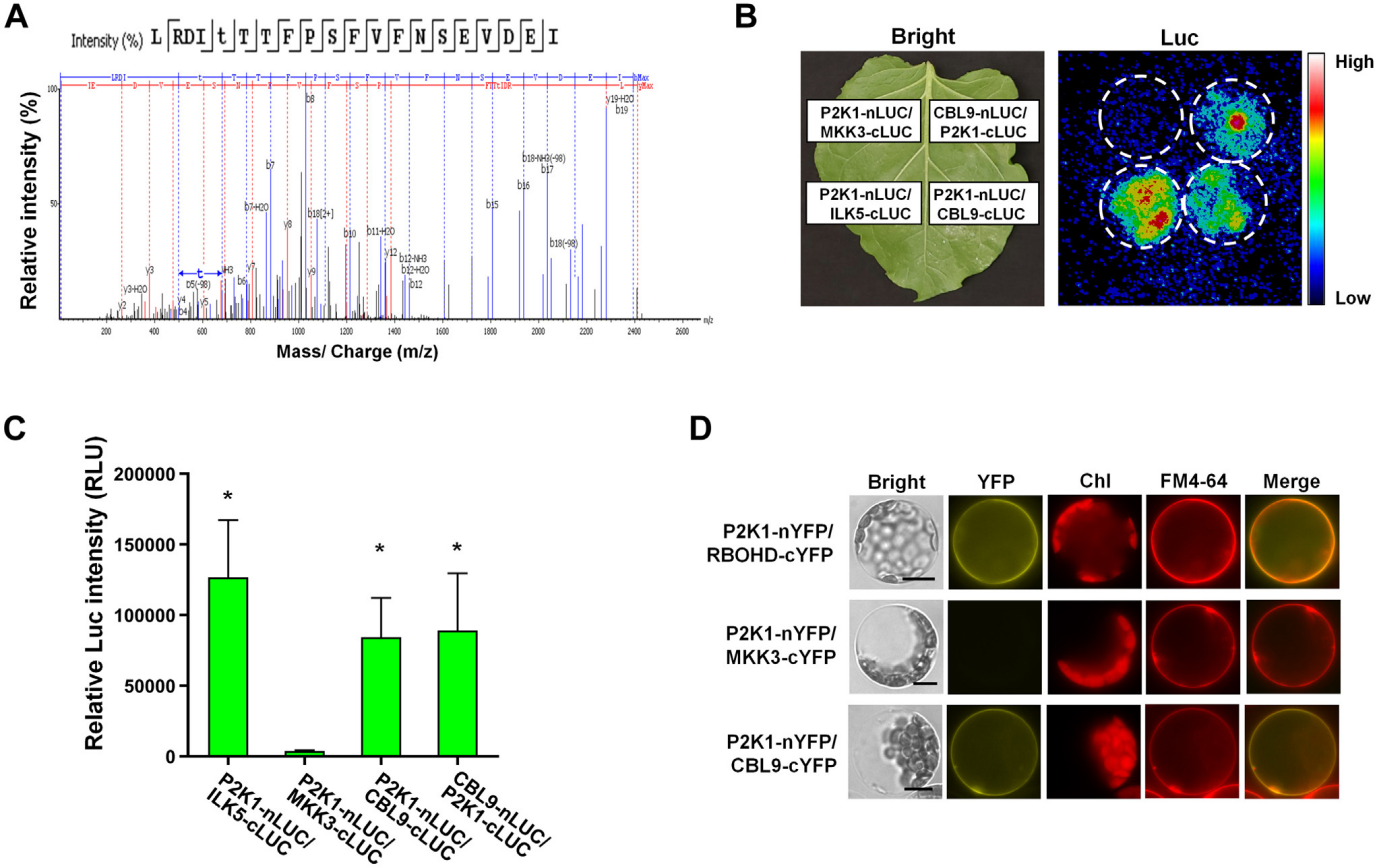
**CBL9 was identified as a P2K1 substrate by KiC assay using the 8k peptide library.***A*, spectrum of phosphopeptide of CBL9. *B*, the interaction between P2K1 and CBL9 was assessed *via* a split-luciferase assay. *Dotted circles* indicate the regions of interest corresponding to the infiltrated areas in *N. benthamiana* leaves. ILK5-cLUC and MKK3-cLUC proteins were employed as positive and negative controls, respectively. *C*, the P2K1–CBL9 interaction signal intensities were quantified. Images captured during the experiment were analyzed using the C-vision/Im32 software to measure luciferase signal intensities. The data was then analyzed using GraphPad Prism 8. The results are expressed as the mean ± SEM, four biological replicates. Statistical significance was determined *via* unpaired two-tailed Student’s *t* test, with levels of significance indicated as follows: ∗∗∗∗*p* < 0.0001, ∗∗∗*p* < 0.001, ∗∗*p* < 0.01, and ∗*p* < 0.05. The *p* values reflect the significance in comparison to the MKK3-cLUC control. *D*, a BiFC assay was conducted in Arabidopsis protoplasts. FM4-64 was used as a plasma membrane marker, and Chl represents chlorophyll autofluorescence. The merged image represents the overlapping fluorescence signals from YFP and FM4-64. Positive and negative controls were provided by RBOHD-cYFP and MKK3-cYFP, respectively. Scale bars represent 10 μm. All experiments were repeated at least twice (biological replicates) with consistent results. BiFC, bimolecular fluorescence complementation; CBL9, calcineurin B–like protein 9; KiC, kinase client; RBOHD, respiratory burst oxidase homolog D.

Given P2K1's presence on the plasma membrane and involvement in stress responses, we focused on potential P2K1 substrates using subcellular localization and spatiotemporal expression resources like SUBA (https://suba.live/) and eFP browser (http://bar.utoronto.ca/efp2/). Furthermore, P2K1 has been implicated in plant physiological processes known to be impacted by eATP signaling, such as cell damage, resistance to pathogens, and touch response, acting as a damage-associated molecular pattern signal receptor (53). As a result, we concentrated on the relevance of the following four candidate substrates: CBL9 (*At5g47100*), GPA1 (*At2g26300*), RING-H2 zinc finger protein ATL6 (ATL6; *At3g05200*), and a leucine-rich repeat RLK protein (LRR-RK; *At5g49770*) (Fig. 6*A* and Supplemental Figs. S8 and S9, *A* and *B*). We initially conducted a split-LCI assay to determine the ability of these candidates to interact with P2K1. The results showed that coinfiltration into *N. benthamiana* leaves of vectors coding for P2K1 and CBL9 and GPA1 (Fig. 6*B* and Supplemental Fig. S8, *B* and *C*), but not ATL6 and LRR-RK, produced clear bioluminescence signals (Supplemental Fig. S9, *C* and *D*). Quantification revealed that the signals were highly significant for CBL9 and GPA1 (Fig. 6*C* and Supplemental Fig. S8*C*). Furthermore, coexpression of P2K1-nYFP with CBL9-cYFP produced a yellow fluorescent signal that colocalized with the plasma membrane marker FM4-64, whereas coexpression of P2K1-nYFP with the control MKK3-cYFP protein did not display a yellow fluorescent signal (Fig. 6*D*). These results indicate that P2K1 interacts with CBL9 and GPA1, highlighting their potential as putative substrates of P2K1.

### P2K1 Kinase Protein Directly Phosphorylates CBL9 and GPA1

**Fig. 7:**
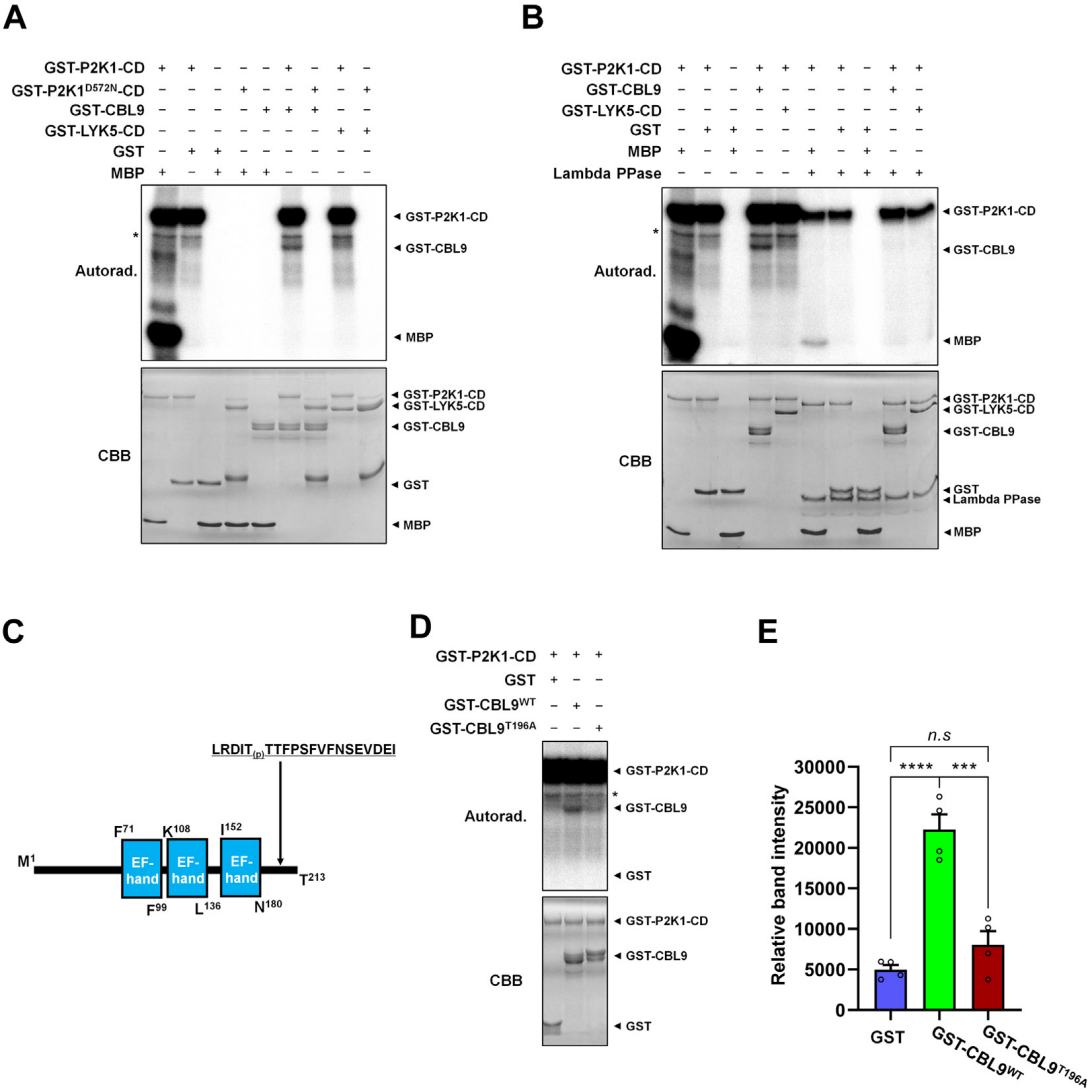
**CBL9 is phosphorylated by P2K1 kinase protein.***A*, GST-P2K1-CD protein directly phosphorylates GST-CBL9, whereas GST-LYK5-CD is not phosphorylated *in vitro*. Bacterial recombinant GST-CBL9 protein was incubated with GST-tagged P2K1 cytosolic domain (GST-P2K1-CD, GST-P2K1^D572N^-CD; a kinase-dead version), or GST. *B*, phosphorylation of CBL9 was confirmed by treatment with Lambda protein phosphatase (Lambda PPase), which releases phosphate groups from phosphorylated serine, threonine, and tyrosine residues. *C*, a schematic diagram illustrates the structural features of CBL9, including the three EF-hand motifs (calcium-binding domain). The identified phosphopeptide LRDIT(p)TTFPSFVFNSEVDEI is located in the C-terminal domain of CBL9. *D* and *E*, mutation of CBL9 at the residue T196 led to reduced phosphorylation by P2K1 *in vitro*. The data are presented as mean ± SEM (n = 4), based on measurements from four independent experiments. Significance levels are indicated as ∗∗*p* < 0.01, with the *p* value indicating significance concerning the band intensity of GST-CBL9^WT^, as determined by unpaired two-tailed Student’s *t* test. In panels (*A*, *B*, and *D*), autophosphorylation and transphosphorylation events were identified through the incorporation of [γ-^32^P] ATP. Myelin basic protein (MBP) was used as a universal substrate, and GST-LYK5-CD was used as a negative control. Protein loading was visualized with Coomassie Brilliant Blue (CBB) staining. The *asterisk* (∗) indicates nonspecific signals. These experiments were conducted at least twice and consistently yielded similar results. CBL9, calcineurin B–like protein 9; CD, cytosolic domain; GST, glutathione-*S*-transferase.

To confirm the KiC results, purified recombinant CBL9 and GPA1 protein were incubated with either purified GST-P2K1-CD, kinase dead version of P2K1 (GST-P2K1^D572N^-CD) in the presence of [γ-^32^P] ATP. The results of this assay showed that GST-P2K1-CD directly trans-phosphorylated GST-CBL9, His-GPA1 as well as the generic substrate myelin basic protein, but not GST-LYK5-CD protein (14, 50) used as a negative control. Assays performed with the kinase-dead versions of P2K1 also failed to phosphorylate GST-CBL9 or His-GPA1 (Fig. 7*A* and Supplemental Fig. S8*D*).

Verifying that the radiography signal was the result of phosphorylation, the addition of lambda protein phosphatase was shown to reduce both autophosphorylation and transphosphorylation of P2K1 and CBL9 or GPA1 protein (Fig. 7*B* and Supplemental Fig. S8*E*). MS/MS results from the KiC assay revealed that the Thr196 residue of CBL9 and Ser52 residue of GPA1 was the specific target of P2K1 phosphorylation (Fig. 7*C* and Table 2). To confirm this, site-directed mutagenesis was used to generate a GST-CBL9^T196A^ mutated protein, which was purified (*e.g.*, the intact GPA1 protein with the Ser52 mutated to A was unsuccessful in purification) and incubated with purified recombinant GST-P2K1-CD protein. As expected, the results showed a significant reduction in phosphorylation of the GST-CBL9^T196A^ recombinant protein, compared to the WT protein (Fig. 7, *D* and *E*). Thus, this example of CBL9 and GPA1 demonstrates the utility of the 8k KiC library to expand the list of P2K1 kinase substrates and to accurately predict the specific site of phosphorylation.

**Table 2:** KiC assay results utilizing the 8k peptide library reveal a putative candidate list of plasma membrane–localized proteins phosphorylated by P2K1 kinase

Arabidopsisrabidopsis Locus ID	Peptide	Probability score	Mass	*m/z*	Phosphopeptide Spectrum count	Stoichiometry (%)^a^	Description
At1g71010	PKNASPT(+79.97)IVSPKQYKRRFRK	111.91	2480.3528	621.0966	11	100	Forms Aploid and binucleate cells 1C
At5g46570	LPKNT(+79.97)VILPTMLSPLGKACA	82.57	2146.1294	716.3882	20	80	BR-signaling kinase 2 (BSK2)
At3g05200	WKLNRT(+79.97)NSLLVLPRGGSSRR	100.6	2389.2856	598.3315	8	73	RING-H2 zinc finger protein ATL6 (ATL6)
At2g45560	GSLNT(+79.97)VVIASPEAAREVLRT	115.02	2162.1096	721.7151	7	70	Cytochrome P450 monooxygenase
At2g26300	KLLLLGAGESGKS(+79.97)TIFKQIK	142.75	2210.2439	737.7588	9	69	G protein alpha subunit 1; GPA1
At1g69420	KKKPQPVKISPWT(+79.97)LARLNAE	131.35	2383.314	795.4484	10	67	DHHC-type zinc finger family protein
At1g16010	DSRRLDKS(+79.97)LSIARSRHDSAR	61.9	2405.2036	482.0513	3	60	Magnesium transporter 2
At5g49770	LLDTT(+79.97)IIQNSGNLKGFEKYV	75.36	2332.1716	778.402	8	57	Leucine-rich repeat protein kinase family protein (LRR-RK)
At2g34690	VNSSAPLITYLDNLFLS(+79.97)KQL	164.78	2315.1814	1158.6056	5	56	Glycolipid transfer protein (GLTP) family protein
At4g24290	GFAPKSGLISTLIS(+79.97)HHFTAA	126.28	2134.0613	712.3712	7	54	MAC/Perforin domain–containing protein
At5g04550	FIGRSHSVS(+79.97)TILTPVSHKSE	90.66	2261.1206	754.7177	8	53	Protein of unknown function (DUF668)
At5g49770	LLDT(+79.97)TIIQNSGNLKGFEKYV	81.08	2332.1716	778.4034	6	50	Leucine-rich repeat protein kinase family protein
At5g47100	LRDIT(+79.97)TTFPSFVFNSEVDEI	200	2409.114	1205.5691	5	50	Calcineurin B–like protein 9 (CBL9)
At3g49190	IKGDFLSTKS(+79.97)KQLRLVHRTV	83.37	2405.3308	482.077	4	50	O-acyltransferase (WSD1-like) family protein
At2g24610	NMQTYLQS(+79.97)ITVRLEEWRLKR	58.1	2643.3357	661.8441	3	50	Cyclic nucleotide–gated channel 14
At1g75640	LKSLSVLNISGCGLT(+79.97)GRIPV	77.88	2106.1272	703.0529	6	46	Leucine-rich receptor-like protein kinase family protein
At2g17220	KLGPSASQSHIT(+79.97)TRVMGTHG	169.25	2144.0198	715.684	6	46	Protein kinase superfamily protein
At4g12420	PQDEFDKT(+79.97)FSMNQARSIRWN	99.31	2549.1157	638.2895	11	44	Cupredoxin superfamily protein
At1g23020	IWIMMPT(+79.97)STYKKIWLKSMRA	114.84	2563.2917	641.8333	6	43	Ferric reduction oxidase 3
At3g13530	QVLVKQMAT(+79.97)SLLKALHINTI	95.44	2300.269	767.7698	2	40	Mitogen-activated protein kinase kinase kinase 7
At5g42750	VKTKPIKS(+79.97)FSLFGLSKWRKG	128.66	2386.3289	597.5954	5	38	BRI1 kinase inhibitor 1
At3g26600	KSSKS(+79.97)NVYRDIGGSGSRTGN	75.73	2148.9912	538.2593	3	38	Armadillo repeat only 4; Armadillo repeat protein
At4g18910	LREITKS(+79.97)GSFLKTVRNGSSR	151.56	2315.2109	579.813	3	38	NOD26-like intrinsic protein 1
At2g36910	THT(+79.97)QVIGMTSGSSSRVKEDD	129.89	2213.9624	738.9961	4	36	ABC subfamily B1
At3g46680	ALNMKGFSIT(+79.97)VVEGQFNKVS	146.35	2248.0962	750.3759	6	33	UDP-glycosyltransferase superfamily protein
At2g41705	LGKDSQNLRWVPFGT(+79.97)LIANV	68.92	2307.1777	770.0717	4	31	Camphor resistance CrcB family protein
At5g49070	TSSSS(+79.97)IWYALSYLEAKRRMK	87.46	2456.1924	819.7463	3	30	3-ketoacyl-CoA synthase 21 (KCS21)
At1g71090	RVFNSISSFS(+79.97)QTSFPEVDLG	200	2296.0413	1149.031	7	28	Auxin efflux carrier family protein
At2g35350	SNRFISPFPSDRFVSPT(+79.97)ASF	115.49	2338.0784	780.3698	5	28	Poltergeist like 1
At4g32300	GVVTSSSLLGNS(+79.97)WRFFDQKQ	139.13	2335.0999	779.3777	8	27	S-domain-2 5
At3g10980	NGLFT(+79.97)LMCLYQHPKRFYHLV	78.82	2559.2319	640.8193	8	26	PLAC8 family protein
At3g46680	ALNMKGFS(+79.97)ITVVEGQFNKVS	92.98	2248.0962	750.3729	4	25	UDP-glycosyltransferase superfamily protein
At4g24290	GFAPKSGLIS(+79.97)TLISHHFTAA	126.99	2134.0613	712.3658	2	25	MAC/Perforin domain–containing protein
At1g47900	IFVLGKQLKS(+79.97)FRPQPEQMRS	116.31	2468.2764	823.7696	4	22	Plant protein of unknown function (DUF869)
At1g28440	MERSKWT(+79.97)LMSFHKLGFSEHE	62.39	2559.144	640.7952	2	22	HAESA-like 1
At5g47070	SET(+79.97)SSFNLQTPRSLPSPRSI	104.91	2283.0896	762.0419	2	22	Protein kinase superfamily protein
At4g12420	PQDEFDKTFSMNQARS(+79.97)IRWN	56.19	2549.1157	850.7167	2	13	Cupredoxin superfamily protein

KiC, kinase client.

### *In Vivo* Phosphoproteomics Screens of *p2k1-3* Knockout and *P2K1*-Overexpression Lines as an Alternative Approach to Identify Kinase Substrates

In addition to the KiC assay, we attempted to identify P2K1 kinase substrates through differential phosphoproteomic screens. By analyzing intensity values from *in vivo* phosphoproteomics data of P2K1 mutant and overexpression lines, we could assemble a list of phosphopeptides that were enriched between the knockout and overexpression line and thus potential targets for P2K1, an indirect but complementary approach to the KiC assay. The two analysis strategies for the phosphoproteomics screen were based on (1): intensity values and (2) intensity values associated with fold change. Following the first approach (P2K1 mutant (*p2k1-3*) < WT (Col-0) < *P2K1-*overexpression line), mock samples showed 1216 phosphopeptides and ATP-treated samples showed 3243 phosphopeptides (Supplemental Table S6*A*). When compared to the 177 KiC assay candidates, 17 gene accessions overlapped with the mock samples and 43 accessions overlapped with the ATP-treated samples (Supplemental Table S6*B*). Second, when adopting the intensity values combined with fold change (P2K1 mutant (*p2k1-3*) < WT (Col-0) (fold change >1.5) < *P2K1-*overexpression line (fold change >1.5)), mock samples included 180 phosphopeptides and ATP-treated samples included 440 phosphopeptides, wherein 3 and 9 gene accession overlapped with the 177 KiC assay candidates, respectively.

## Discussion

### ILK4 May Function as a Negative Regulator of MAPK Cascades

In our previous study, we identified ILK5 as a positive regulator in the MAPK cascade, which is phosphorylated at the Ser192 residue by P2K1 in response to pathogen invasion (33). This phosphorylation event triggers MAPK activation, leading to the initiation of plant innate immunity (33). In the present study, we report the phosphorylation of ILK4, which, like ILK5, interacts with P2K1 and participates in protein–protein interactions with MKK4 and MKK5 (Fig. 2). Interestingly, in the *ilk5-1* mutant, phosphorylation of MPK3 and MPK6 was significantly reduced under ATP treatment, whereas in the *ilk4-1* mutant, phosphorylation levels of MPK3 and MPK6 were elevated compared to the WT Col-0 (Fig. 2). The molecular basis for this difference is hinted at by the KiC assay results, which revealed that the P2K1 phosphorylation site on ILK4 and ILK5 are far apart in the protein sequences. Analysis with the Plant PTM viewer (https://www.psb.ugent.be/webtools/ptm-viewer/index.php) reveals that Ser192 of ILK5 is phosphorylated, whereas the corresponding ILK4 does not have any phosphorylated residues in the nearby position. Instead, Ser330 and Ser331 of ILK4 are phosphorylated in the plant PTM viewer, but the corresponding phosphorylated residues in ILK5 are not present. Furthermore, gene expression analysis demonstrated that ILK5 is strongly induced by a variety of pathogens, the elicitor flg22, and several stress conditions (33). In contrast, ILK4 expression was induced in response to wounding, with minimal changes observed under other pathogen or stress conditions in the eFP browser (https://bar.utoronto.ca/efp2/). Furthermore, ILK5 showed increased expression throughout the whole plant, particularly around the stomata (33), whereas ILK4 exhibited weak expression concentrated in the leaf veins (Supplemental Fig. S2*C*).

The functionality of animal or plant ILK proteins as true kinases remains a topic of debate due to substitutions of several key residues that are critical for kinase activity (33, 51, 54). Notably, ILK5 displayed robust kinase activity when purified from plant tissues (33). In contrast, structural analysis revealed a key difference in the kinase domain of ILK4 relative to ILK5. The HRDL consensus sequence in the C-loop VIb of ILK5, which is essential for kinase activity, is substituted with a C residue in ILK4, replacing the conserved R residue (33, 51). This substitution suggests that ILK4 may possess reduced kinase activity or potentially function as a pseudo-kinase, competitively interacting with ILK5. This divergent pattern of phosphorylation, along with the differences in gene expression levels or the structure of the protein and the phosphorylated residues, suggests that ILK4 may function as a negative regulator within the MAPK cascade, potentially serving as a fine-tuning mechanism to modulate immune responses and prevent excessive activation.

### The 8k Peptide Library Expansion Enabled the Identification of Novel Substrates for the P2K1 Purinoreceptor

The identification of substrates for kinases is crucial for understanding intracellular signaling and regulation of protein activity by phosphorylation of Ser, Thr, and Tyr residues. The KiC assay was first introduced by Huang *et al*. 2010 (7) and further developed by Ahsan *et al*. (9). This assay involves *in vitro* kinase assays with recombinant, purified kinase proteins, and synthetic peptides designed from *in vivo* phosphopeptides. Phosphorylated peptides can then be analyzed in a high-throughput manner by LC-MS/MS. Subsequently, the KiC assay method was successfully applied to analyze the P2K1 RLK (7, 14, 33, 34). The results identified an NADPH oxidase, RBOHD, as an interacting partner of P2K1 kinase (14). This finding revealed that purinergic signaling mediated by eATP induces rapid synthesis of reactive oxygen species through the phosphorylation of RBOHD's N-terminal Ser22 and Thr24, leading to stomatal closure (Supplemental Fig. S1*A*) (14). This reactive oxygen species-induced stomatal closure mechanism plays a critical role in the defense against the invasion of plant tissues by bacterial pathogens, such as *P. syringae* (14). In another set of results from the KiC assay, it was established that the phosphorylation of PAT5 and PAT9 regulates the activity of the P2K1 protein (34). Specifically, it was shown that the S-acylation process prevents excessive activation of P2K1 and regulates its turnover (34). More recently, P2K1 was shown to regulate phosphorylation of Ser192 of ILK5, a Raf-like MAPKKK, which in turn controls the phosphorylation of MPK3/6 in plants and contributes to stomatal immunity (Supplemental Fig. S1*A*) (33).

Here, we identify and verify two additional P2K1 protein interactors from the list of candidates identified in the 2k and 8k peptide library screenings. We expanded the peptide library from 2k to 8k, allowing for the identification of a broader range of kinase substrates. In particular, the KiC assay using the 8k peptide library in this study identified 177 putative substrates (Supplemental Tables S3 and S8). Among them, the protein–protein interaction between P2K1 and CBL9 or GPA1 within the plant system was verified by various biochemical and molecular biological validation methods (Figs. 6 and 7 and Supplemental Fig. S8). Previous experience with the 2k library showed that approximately 50% of the peptides predicted by the KiC assay to be substrates for the P2K1 kinase could be subsequently confirmed using the types of assays described above for CBL9 and GPA1. Kinase–substrate interactions can be transient and, therefore, the assays used may not be suitable in all cases to confirm the interactions predicted by the KiC assay. However, even at ∼50%, the KiC assay provides a wealth of information about putative kinase substrates that can be further pursued depending on the specific biological question of interest. Lastly, as a final selection criteria to narrow down to the most likely biological interactions, we relied on a) subcellular localization information (plasma membrane and cytoplasm for the P2K1 kinase), b) biological function (pathogen defense, wounding, touch response, and plant growth), and c) similar gene expression patterns at the mRNA level between the kinase and the candidate protein.

### Analysis of CBL9 and GPA1 Function in the Context of Purinergic Signaling within the Plant System

Previous reports showed that eATP causes a rapid increase in intracellular calcium concentration, which is dependent on the P2K1 purinoreceptor (24). Presumably this rise in calcium would then be sensed in the cytoplasm by specific calcium-binding proteins, whose activity is known to be regulated *via* intracellular signaling cascades (1, 55, 56). Notably, CBL9 was reported to be highly calcium-dependent as a calcium-binding protein, and it is known to physically interact with CIPK3 or CIPK23, thereby regulating the kinase activity of CIPK3 or CIPK23 (57, 58, 59). Recent reports highlighted the involvement of CBL9 in abscisic acid (ABA) signaling and ABA synthesis, which in turn regulate plant growth in response to osmotic stress (58). In a previous report, CBL1/9–CIPK23 complex phosphorylates the K^+^ channel AKT1, playing a role in K^+^ uptake under conditions of limited K^+^ supply. Interestingly, the phosphorylation of serine 201 in CBL1 and CBL9 is crucial for modulating the activity of AKT1 (60). Our KiC data shows that the CBL9 threonine 196 residue, adjacent to serine 201, is a target for the P2K1 kinase (Fig. 7). Therefore, it is quite possible that phosphorylation of this region of CBL9, either by CIPK23 or P2K1, is a key event in the regulation of CBL9 activity, perhaps *via* interaction with other proteins. This may be particularly relevant to the rapid inhibition of plant growth induced by salt stress, which is associated with the extracellular accumulation of eATP and its perception through P2K1-mediated purinergic signaling (31). Interestingly, CBL9, in conjunction with CBL1, plays a significant role in pollen tube germination and the elongation of germinated pollen tubes (57). This is consistent with recent findings indicating that eATP-mediated purinergic signaling also plays a pivotal role in pollen tube germination and growth (61, 62). These observations suggest that the P2K1 purinoreceptor directly phosphorylates CBL9, ultimately influencing pollen tube germination and growth under stress conditions.

GPA1 plays a crucial role in regulating various plant biological processes, such as inhibiting the opening of stomata in response to ABA hormone regulation to control transpiration efficiency, growth inhibition under stress conditions, and plant defense mechanisms against pathogens etc (63, 64, 65). Furthermore, it was reported to play a significant role in eATP signaling triggered by mechanical stimuli, such as touch (20). Interestingly, it was suggested that GPA1 may regulate thigmomorphogenesis through direct interaction with P2K1 in response to touch. Additionally, in animals, P2Y functions as a GPCR, directly binding ATP to mediate signal transduction as an essential purinergic receptor (66). Therefore, investigating the interaction between GPCRs and G proteins in plants, particularly in relation to P2K1, an eATP receptor, presents a fascinating research topic for the future.

### Overlap Verification of Phosphopeptide Candidates

A comprehensive overlap verification study of 177 phosphopeptide candidates at the protein accession level revealed a 45% overlap with *in vivo* phosphoproteomic datasets on P2K1 lines (Supplemental Table S7*A*). Significant variation in overlap can be observed depending on the phosphoproteomics data analysis adopted (Supplemental Table S7*B*). Furthermore, the analysis of intensity values from *in vivo* phosphoproteomics data highlights the limitations of indirect approaches, such as phosphoproteomics screens, in discovering kinase clients, as high background phosphorylation hinders the reliable identification of specific kinase targets.

The detection of phosphorylation sites in phosphoproteomics is often influenced by several factors. For instance, the low abundance and substoichiometric nature of phosphorylated proteins can lead to missed sites due to sensitivity constraints of mass spectrometry techniques (67). Furthermore, biological sample variability, including differences in cell lines and experimental conditions (68), as well as variation in site identification criteria, quantification thresholds, and protease digestion strategies (69) can significantly impact the phosphorylation landscape, leading to divergent results. Lastly, the use of different mass spectrometry techniques and data analysis pipelines can further complicate comparisons and result in discrepancies in false localization and data interpretation (70, 71). Therefore, the KiC assay can also help elucidate such discrepancies, especially at the phosphorylation site level (Supplemental Table S8), where higher variability can be observed in different *in vivo* phosphoproteomics studies.

### The Advantages and Potential Advancements of the KiC Assay in the Discovery of Substrates for Various Kinases

The KiC assay provides several advantages for the discovery of kinase substrates (14, 33, 34). It excels in high-throughput analysis, allowing efficient screening of numerous peptides, providing specificity in identifying kinase–substrate interactions, high sensitivity, and low signal-to-noise ratio. Its versatility allows it to adapt to various kinases and peptides, making it a valuable tool for exploring diverse kinase-substrate relationships. The inclusion of validation through biochemical and molecular biology techniques increases the reliability of results. The KiC assay also provides valuable insight into signal transduction pathways by elucidating the modulation of cellular responses by kinases. The KiC assay has its limitations, including that its operation *in vitro* requires purification of protein kinase and does not fully simulate the complex interactions that occur in the cellular environment. Additionally, the analysis and synthesis of a large peptide library can be costly, and other PTMs (*e.g.*, ubiquitination, acylation, etc) are not addressed. Furthermore, the KiC assay only includes peptides represented in the library, leading to potential limitations in the results.

Several strategies can be employed to mitigate these limitations. First, integrating KiC assay results with a hierarchy of *in vivo* experiments can account for the complexity of the intracellular environment. This approach allows for a more comprehensive understanding by considering the dynamic nature of cellular processes. Second, optimizing the peptide library can improve the accuracy of the analysis including quantitative information of PMS counts and stoichiometry. This optimization not only contributes to the provision of quantitative details regarding PMS counts and stoichiometry but also guarantees a more accurate and reliable outcome overall.

Looking ahead, the potential for further development of the KiC assay is promising. Further expanding the size of the peptide libraries may increase the likelihood of discovering novel kinase substrates. Integration with proteomics techniques can provide a comprehensive view of the kinome and its substrates. Automation can also be employed to streamline the process, increasing efficiency and throughput. Combining the KiC assay with structural biology modeling (*e.g.*, alpha fold) and techniques may provide insight into the structural basis of kinase–substrate interactions (72). In summary, the strengths of the KiC assay and its potential for future advancements make it a valuable tool in kinase research, facilitating a deeper understanding of cellular processes and signaling pathways with implications in various fields.

## Data Availability

The mass spectrometry proteomics data for this study can be accessed through the ProteomeXchange Consortium using the dataset identifier PXD006678 and PXD056072 in the PRIDE partner repository and MassIVE database, accession MSV000095888. Furthermore, all additional data supporting the results and conclusions of this research are either provided in the article and its supplemental files or can be obtained from the corresponding author upon request.

## Supplemental data

This article contains supplemental data.

## Conflict of interest

The authors declare that the research was conducted in the absence of any commercial or financial relationships that could be construed as a potential conflict of interest.

## References

[1] Ubersax J.A., Ferrell J.E. (2007). Mechanisms of specificity in protein phosphorylation. Nat. Rev. Mol. Cell Biol..

[2] Leutert M., Entwisle S.W., Villén J. (2021). Decoding post-translational modification crosstalk with proteomics. Mol. Cell Proteomics.

[3] Zhang W.J., Zhou Y., Zhang Y., Su Y.H., Xu T. (2023). Protein phosphorylation: a molecular switch in plant signaling. Cell Rep..

[4] Shiu S.H., Bleecker A.B. (2003). Expansion of the receptor-like kinase/Pelle gene family and receptor-like proteins in Arabidopsis. Plant Physiol..

[5] Zulawski M., Schulze G., Braginets R., Hartmann S., Schulze W.X. (2014). The Arabidopsis Kinome: phylogeny and evolutionary insights into functional diversification. BMC Genomics.

[6] Markossian S., Grossman A., Brimacombe K., Arkin M., Auld D., Austin C. (2004).

[7] Huang Y., Houston N.L., Tovar-Mendez A., Stevenson S.E., Miernyk J.A., Randall D.D. (2010). A quantitative mass spectrometry-based approach for identifying protein kinase clients and quantifying kinase activity. Anal. Biochem..

[8] Huang Y., Thelen J.J. (2012). KiC assay: a quantitative mass spectrometry-based approach for kinase client screening and activity analysis [corrected]. Methods Mol. Biol..

[9] Ahsan N., Huang Y., Tovar-Mendez A., Swatek K.N., Zhang J., Miernyk J.A. (2013). A versatile mass spectrometry-based method to both identify kinase client-relationships and characterize signaling network topology. J. Proteome Res..

[10] Faria R., Ferreira L., Bezerra R., Frutuoso V., Alves L. (2012). Action of natural products on p2 receptors: a reinvented era for drug discovery. Molecules.

[11] Ferrari D., McNamee E.N., Idzko M., Gambari R., Eltzschig H.K. (2016). Purinergic signaling during immune cell trafficking. Trends Immunol..

[12] Matzinger P. (2007). Friendly and dangerous signals: is the tissue in control?. Nat. Immunol..

[13] Cekic C., Linden J. (2016). Purinergic regulation of the immune system. Nat. Rev. Immunol..

[14] Chen D., Cao Y., Li H., Kim D., Ahsan N., Thelen J. (2017). Extracellular ATP elicits DORN1-mediated RBOHD phosphorylation to regulate stomatal aperture. Nat. Commun..

[15] Song C.J., Steinebrunner I., Wang X., Stout S.C., Roux S.J. (2006). Extracellular ATP induces the accumulation of superoxide *via* NADPH oxidases in Arabidopsis. Plant Physiol..

[16] Deng S., Sun J., Zhao R., Ding M., Zhang Y., Sun Y. (2015). Populus euphratica APYRASE2 enhances cold tolerance by modulating vesicular trafficking and extracellular ATP in Arabidopsis plants. Plant Physiol..

[17] Thomas C., Rajagopal A., Windsor B., Dudler R., Lloyd A., Roux S.J. (2000). A role for ectophosphatase in xenobiotic resistance. Plant Cell.

[18] Lew R.R., Dearnaley J.D.W. (2000). Extracellular nucleotide effects on the electrical properties of growing Arabidopsis thaliana root hairs. Plant Sci..

[19] Zhu R., Dong X., Hao W., Gao W., Zhang W., Xia S. (2017). Heterotrimeric G protein-regulated Ca2+ Influx and PIN2 asymmetric distribution are involved in Arabidopsis thaliana roots’ avoidance response to extracellular ATP. Front. Plant Sci..

[20] Weerasinghe R.R., Swanson S.J., Okada S.F., Garrett M.B., Kim S.-Y., Stacey G. (2009). Touch induces ATP release in Arabidopsis roots that is modulated by the heterotrimeric G-protein complex. FEBS Lett..

[21] Tang W., Brady S.R., Sun Y., Muday G.K., Roux S.J. (2003). Extracellular ATP inhibits root gravitropism at concentrations that inhibit polar auxin transport. Plant Physiol..

[22] Chivasa S., Ndimba B.K., Simon W.J., Lindsey K., Slabas A.R. (2005). Extracellular ATP functions as an endogenous external metabolite regulating plant cell viability. Plant Cell.

[23] Feng H., Guan D., Bai J., Sun K., Jia L. (2015). Extracellular ATP: a potential regulator of plant cell death. Mol. Plant Pathol..

[24] Choi J., Tanaka K., Cao Y., Qi Y., Qiu J., Liang Y. (2014). Identification of a plant receptor for extracellular ATP. Science.

[25] Choi J. (2013).

[26] Bouwmeester K., de Sain M., Weide R., Gouget A., Klamer S., Canut H. (2011). The lectin receptor kinase LecRK-I.9 is a novel Phytophthora resistance component and a potential host target for a RXLR effector. PLoS Pathog..

[27] Balagué C., Gouget A., Bouchez O., Souriac C., Haget N., Boutet-Mercey S. (2017). The Arabidopsis thaliana lectin receptor kinase LecRK-I.9 is required for full resistance to Pseudomonas syringae and affects jasmonate signalling. Mol. Plant Pathol..

[28] Bouwmeester K., Han M., Blanco-Portales R., Song W., Weide R., Guo L.-Y. (2014). The Arabidopsis lectin receptor kinase LecRK-I.9 enhances resistance to Phytophthora infestans in Solanaceous plants. Plant Biotechnol. J..

[29] Wang Y., Nsibo D.L., Juhar H.M., Govers F., Bouwmeester K. (2016). Ectopic expression of Arabidopsis L-type lectin receptor kinase genes LecRK-I.9 and LecRK-IX.1 in Nicotiana benthamiana confers Phytophthora resistance. Plant Cell Rep..

[30] Pham A.Q., Cho S.-H., Nguyen C.T., Stacey G. (2020). Arabidopsis lectin receptor kinase P2K2 is a second plant receptor for extracellular ATP and contributes to innate immunity. Plant Physiol..

[31] Kim D., Yanders S., Stacey G. (2023). Salt stress releases extracellular ATP to activate purinergic signaling and inhibit plant growth. Plant Physiol..

[32] Kim S.-Y., Sivaguru M., Stacey G. (2006). Extracellular ATP in plants. Visualization, localization, and analysis of physiological significance in growth and signaling. Plant Physiol..

[33] Kim D., Chen D., Ahsan N., Jorge G.L., Thelen J.J., Stacey G. (2023). The Raf-like MAPKKK INTEGRIN-LINKED KINASE 5 regulates purinergic receptor-mediated innate immunity in Arabidopsis. Plant Cell.

[34] Chen D., Hao F., Mu H., Ahsan N., Thelen J.J., Stacey G. (2021). S-acylation of P2K1 mediates extracellular ATP-induced immune signaling in Arabidopsis. Nat. Commun..

[35] Hayashi M., Inoue S.-I., Ueno Y., Kinoshita T. (2017). A Raf-like protein kinase BHP mediates blue light-dependent stomatal opening. Sci. Rep..

[36] Kilburn R., Fedosejevs E.T., Mehta D., Soleimani F., Ghahremani M., Monaghan J. (2023). Substrate profiling of the Arabidopsis Ca2+-dependent protein kinase AtCPK4 and its Ricinus communis ortholog RcCDPK1. Plant Sci..

[37] Clough S.J., Bent A.F. (1998). Floral dip: a simplified method for Agrobacterium-mediated transformation of Arabidopsis thaliana. Plant J..

[38] Yao Q., Ge H., Wu S., Zhang N., Chen W., Xu C. (2014). P^3^DB 3.0: from plant phosphorylation sites to protein networks. Nucleic Acids Res..

[39] Nielsen F. (2021). On a variational definition for the Jensen-Shannon symmetrization of distances based on the information radius. Entropy (Basel).

[40] Capra J.A., Singh M. (2007). Predicting functionally important residues from sequence conservation. Bioinformatics.

[41] Gao J., Thelen J.J., Dunker A.K., Xu D. (2010). Musite, a tool for global prediction of general and kinase-specific phosphorylation sites. Mol. Cell Proteomics.

[42] Yao Q., Gao J., Bollinger C., Thelen J.J., Xu D. (2012). Predicting and analyzing protein phosphorylation sites in plants using musite. Front. Plant Sci..

[43] Potter S.C., Luciani A., Eddy S.R., Park Y., Lopez R., Finn R.D. (2018). HMMER web server: 2018 update. Nucleic Acids Res..

[44] Hooper C.M., Castleden I.R., Tanz S.K., Aryamanesh N., Millar A.H. (2017). SUBA4: the interactive data analysis centre for Arabidopsis subcellular protein locations. Nucleic Acids Res..

[45] Berardini T.Z., Mundodi S., Reiser L., Huala E., Garcia-Hernandez M., Zhang P. (2004). Functional annotation of the Arabidopsis genome using controlled vocabularies. Plant Physiol..

[46] Zhou L., Zhou M., Gritsenko M.A., Stacey G. (2020). Selective enrichment coupled with proteomics to identify S-acylated plasma membrane proteins in Arabidopsis. Curr. Protoc. Plant Biol..

[47] Cock P.J.A., Antao T., Chang J.T., Chapman B.A., Cox C.J., Dalke A. (2009). Biopython: freely available Python tools for computational molecular biology and bioinformatics. Bioinformatics.

[48] Hwang S.M., Kim D.W., Woo M.S., Jeong H.S., Son Y.S., Akhter S. (2014). Functional characterization of Arabidopsis HsfA6a as a heat-shock transcription factor under high salinity and dehydration conditions. Plant Cell Environ..

[49] Ahsan N., Swatek K.N., Zhang J., Miernyk J.A., Xu D., Thelen J.J. (2012). “Scanning mutagenesis” of the amino acid sequences flanking phosphorylation site 1 of the mitochondrial pyruvate dehydrogenase complex. Front. Plant Sci..

[50] Kim D., Stacey G. (2023). Phosphorylation-mediated regulation of integrin-linked kinase 5 by purinoreceptor P2K2. Plant Signal. Behav..

[51] Popescu S.C., Brauer E.K., Dimlioglu G., Popescu G.V. (2017). Insights into the structure, function, and ion-mediated signaling pathways transduced by plant integrin-linked kinases. Front. Plant Sci..

[52] Cho S.-H., Tóth K., Kim D., Vo P.H., Lin C.-H., Handakumbura P.P. (2022). Activation of the plant mevalonate pathway by extracellular ATP. Nat. Commun..

[53] Choi J., Tanaka K., Liang Y., Cao Y., Lee S.Y., Stacey G. (2014). Extracellular ATP, a danger signal, is recognized by DORN1 in Arabidopsis. Biochem. J..

[54] Brauer E.K., Ahsan N., Dale R., Kato N., Coluccio A.E., Piñeros M.A. (2016). The Raf-like kinase ILK1 and the high affinity K+ transporter HAK5 are required for innate immunity and abiotic stress Response1[OPEN]. Plant Physiol..

[55] Liu X., Zhou Y., Du M., Liang X., Fan F., Huang G. (2022). The calcium-dependent protein kinase CPK28 is targeted by the ubiquitin ligases ATL31 and ATL6 for proteasome-mediated degradation to fine-tune immune signaling in Arabidopsis. Plant Cell.

[56] Tian W., Hou C., Ren Z., Wang C., Zhao F., Dahlbeck D. (2019). A calmodulin-gated calcium channel links pathogen patterns to plant immunity. Nature.

[57] Mähs A., Steinhorst L., Han J.-P., Shen L.-K., Wang Y., Kudla J. (2013). The calcineurin B-like Ca2+ sensors CBL1 and CBL9 function in pollen germination and pollen tube growth in Arabidopsis. Mol. Plant.

[58] Pandey G.K., Grant J.J., Cheong Y.H., Kim B.-G., Li L.G., Luan S. (2008). Calcineurin-B-like protein CBL9 interacts with target kinase CIPK3 in the regulation of ABA response in seed germination. Mol. Plant.

[59] Ragel P., Ródenas R., García-Martín E., Andrés Z., Villalta I., Nieves-Cordones M. (2015). The CBL-interacting protein kinase CIPK23 regulates HAK5-mediated high-affinity K+ uptake in Arabidopsis roots. Plant Physiol..

[60] Du W., Lin H., Chen S., Wu Y., Zhang J., Fuglsang A.T. (2011). Phosphorylation of SOS3-like calcium-binding proteins by their interacting SOS2-like protein kinases is a common regulatory mechanism in Arabidopsis1. Plant Physiol..

[61] Wu Y., Yin H., Liu X., Xu J., Qin B., Feng K. (2021). P2K1 receptor, heterotrimeric Gα protein and CNGC2/4 are involved in extracellular ATP-promoted ion Influx in the pollen of Arabidopsis thaliana. Plants (Basel).

[62] Wu Y., Qin B., Feng K., Yan R., Kang E., Liu T. (2018). Extracellular ATP promoted pollen germination and tube growth of Nicotiana tabacum through promoting K+ and Ca2+ absorption. Plant Reprod..

[63] Nilson S.E., Assmann S.M. (2010). The alpha-subunit of the Arabidopsis heterotrimeric G protein, GPA1, is a regulator of transpiration efficiency. Plant Physiol..

[64] Yang S., Jung S., Lee H. (2023). Heterotrimeric G protein-mediated signaling is involved in stress-mediated growth inhibition in Arabidopsis thaliana. Int. J. Mol. Sci..

[65] Brenya E., Trusov Y., Dietzgen R.G., Botella J.R. (2016). Heterotrimeric G-proteins facilitate resistance to plant pathogenic viruses in Arabidopsis thaliana (L.) Heynh. Plant Signal. Behav..

[66] Salmaso V., Jacobson K.A. (2020). Purinergic signaling: impact of GPCR structures on rational drug design. ChemMedChem.

[67] Kettenbach A.N., Gerber S.A. (2011). Rapid and reproducible single-stage phosphopeptide enrichment of complex peptide mixtures: application to general and phosphotyrosine-specific phosphoproteomics experiments. Anal. Chem..

[68] Wang P., Xue L., Batelli G., Lee S., Hou Y.-J., Van Oosten M.J. (2013). Quantitative phosphoproteomics identifies SnRK2 protein kinase substrates and reveals the effectors of abscisic acid action. Proc. Natl. Acad. Sci. U. S. A..

[69] Gilmore J.M., Kettenbach A.N., Gerber S.A. (2012). Increasing phosphoproteomic coverage through sequential digestion by complementary proteases. Anal. Bioanal. Chem..

[70] Zhang Y., Dreyer B., Govorukhina N., Heberle A.M., Končarević S., Krisp C. (2022). Comparative assessment of quantification methods for tumor tissue phosphoproteomics. Anal. Chem..

[71] Locard-Paulet M., Bouyssié D., Froment C., Burlet-Schiltz O., Jensen L.J. (2020). Comparing 22 popular phosphoproteomics pipelines for peptide identification and site localization. J. Proteome Res..

[72] Jumper J., Evans R., Pritzel A., Green T., Figurnov M., Ronneberger O. (2021). Highly accurate protein structure prediction with AlphaFold. Nature.

