# Supplementary material for: Identifying Receptor Kinase Substrates Using an 8000 Peptide Kinase Client Library Enriched for Conserved Phosphorylation Sites
Source: Mol Cell Proteomics. 2025 Feb 7;24(3):100926. doi: 10.1016/j.mcpro.2025.100926 (PMC11952801; doi:10.1016/j.mcpro.2025.100926)
Supplement: Supplemental Information [file mmc9.docx]

**Supplemental information**

Identifying receptor kinase substrates using an 8,000 peptide kinase client library enriched for conserved phosphorylation sites

Authors

Daewon Kim^†^, Gabriel Lemes Jorge^†^, Chunhui Xu, Lingtao Su, Sung-Hwan Cho, Nagib Ahsan, Dongqin Chen, Lijuan Zhou, Marina A. Gritsenko, Mowei Zhou, Jinrong Wan, Ljiljana Pasa-Tolic, Dong Xu, Laura E Bartley, Jay J Thelen* and Gary Stacey*

^†^These authors contributed equally to this work

*For correspondence: thelenj@missouri.edu and staceyg@missouri.edu


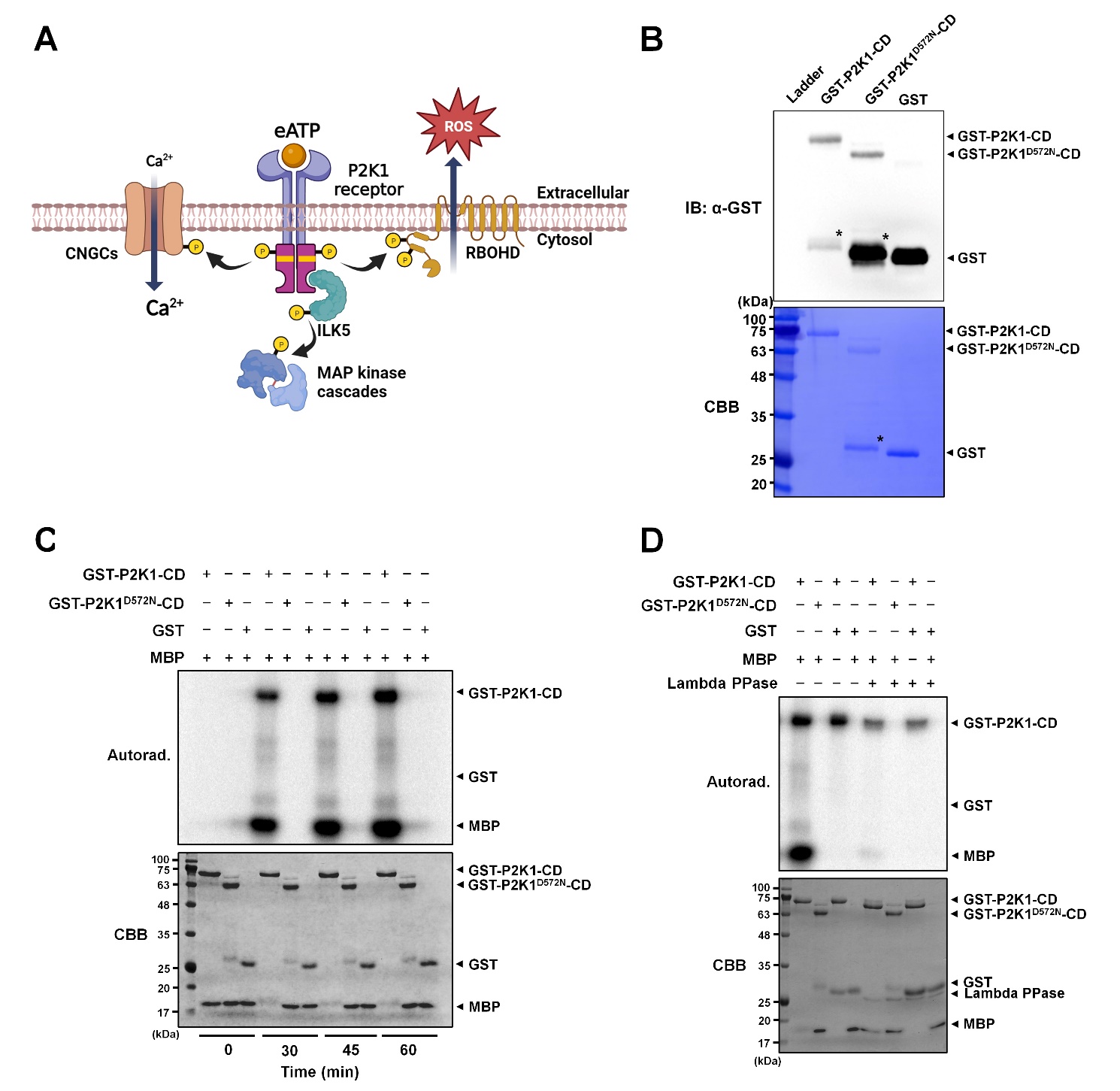


Supplemental Fig. S1. Kinase activity of GST-tagged recombinant P2K1 kinase. *A*, Hypothetical model for the role of P2K1 purinoreceptor in eATP-mediated purinergic signaling cascades. Upon addition of the activating ligand eATP, the P2K1 receptor is rapidly auto-phosphorylated and then interacts with and phosphorylates its downstream targets, such as RBOHD, ILK5 protein etc., leading to a pattern-triggered immune response via a ROS burst, elevation of cytosolic calcium levels and MAPK activation. This figure was created using BioRender (https://biorender.com/). *B*, Detection of purified GST-tagged P2K1 wild-type (GST-P2K1-CD) and a kinase dead version (GST-P2K1^D572N^-CD) of the protein using an anti-GST antibody. “CBB” represents Coomassie Brilliant Blue staining for protein visualization. Asterisk (*) represents the cleaved GST-P2K1 protein. *C*, GST-P2K1-CD protein phosphorylates the MBP universal substrate *in vitro* but not GST protein. Autophosphorylation of P2K1 and transphosphorylation of MBP increase with incubation time (0, 30, 45, and 60 min)*. D*, Phosphorylation of P2K1 and MBP was confirmed by treatment with lambda protein phosphatase (Lambda PPase), which releases phosphate groups from phosphorylated serine, threonine, and tyrosine residues. In panel *C* and *D*, auto- and trans-phosphorylation events were identified through the incorporation of [γ-32P] ATP. Myelin Basic Protein (MBP) was used as a universal substrate, GST was used as a negative control. Protein loading was visualized with Coomassie Brilliant Blue (CBB) staining. All of the above experiments were repeated two times with similar results.


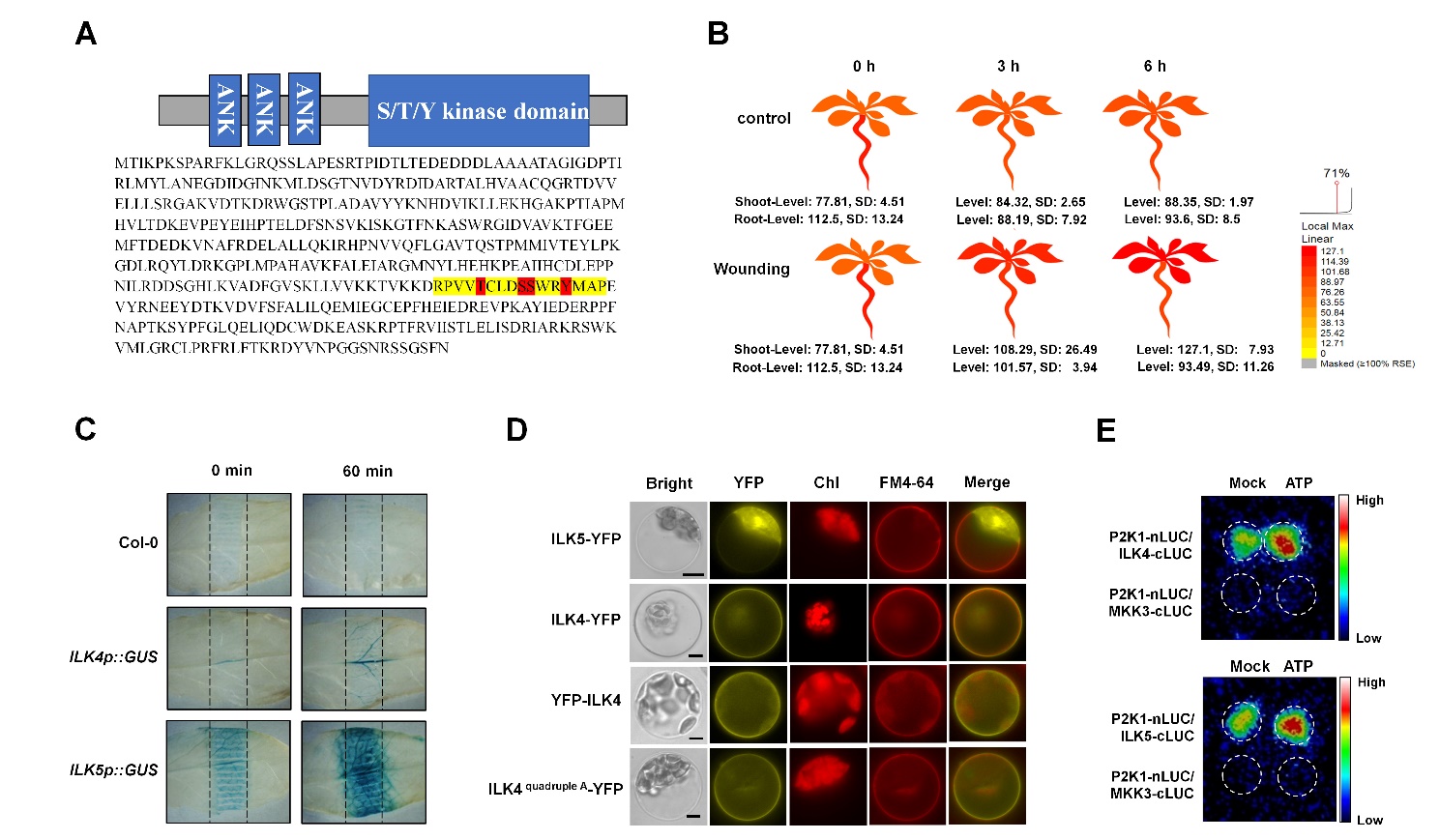


Supplemental Fig. S2. Expression patterns and subcellular localization of ILK4 protein. *A,* Protein sequence of ILK4. In the box, ANK and S/T/Y indicate ankrin repeat and Serine/Threonine/Tyrosine kinase domains, respectively. Yellow and red highlighted regions indicate phosphopeptides and target residues of ILK4 identified in the KiC assay. *B*, Expression of *ILK4* transcripts under wounding stress conditions was analyzed using *in silico* data obtained from BAR ePlant (<http://bar.utoronto.ca/eplant>). *C*, Histochemical analysis of *ILK4promoter::GUS* and *ILK5promoter::GUS* expression in response to wounding. The expression patterns of the *ILK4promoter::GUS* and *ILK5promoter::GUS* transgenic plants were detected by histochemical staining of 2-week-old GUS transgenic plants after wounding. Non-transgenic Col-0 plant were used as a negative control. Dashed lines indicate the wounded area of the rosette leaves. *D*, Subcellular localization of ILK4 and ILK4 ^quadruple A^ proteins in Arabidopsis protoplasts. The fluorescence of ILK4-YFP (C-terminally tagged YFP) or YFP-ILK4 (N-terminally tagged YFP) was monitored 24 h after transformation. FM4-64 was used as a plasma membrane marker. Chl represents chlorophyll auto-fluorescence signal. Merge indicates an overlapped image of YFP and FM4-64. Bars = 10 μm. *E*, Split-LCI experiment showing interaction of the P2K1-ILK4 protein with/without ATP treatment. The split-LCI assay was performed after addition of 200 μM ATP or MES buffer (pH 5.7) as a mock treatment in *N. benthamiana* leaves co-infiltrated with *GV3101* expressing P2K1-nLUC and ILK4-cLUC or ILK5-cLUC. A solution of 1 mM D-luciferin containing 0.01% (v/v) Triton X-100 was sprayed onto the *N. benthamiana* leaves and immediately placed in dark conditions for 10 min to quench the fluorescence. The luminescence was monitored and captured using a low light imaging CCD camera (Photek; Photek, Ltd.). Dotted circles indicate the infiltrated area in *N. benthamiana* leaves. ILK5-cLUC and MKK3-cLUC proteins were used as a positive and a negative control, respectively.


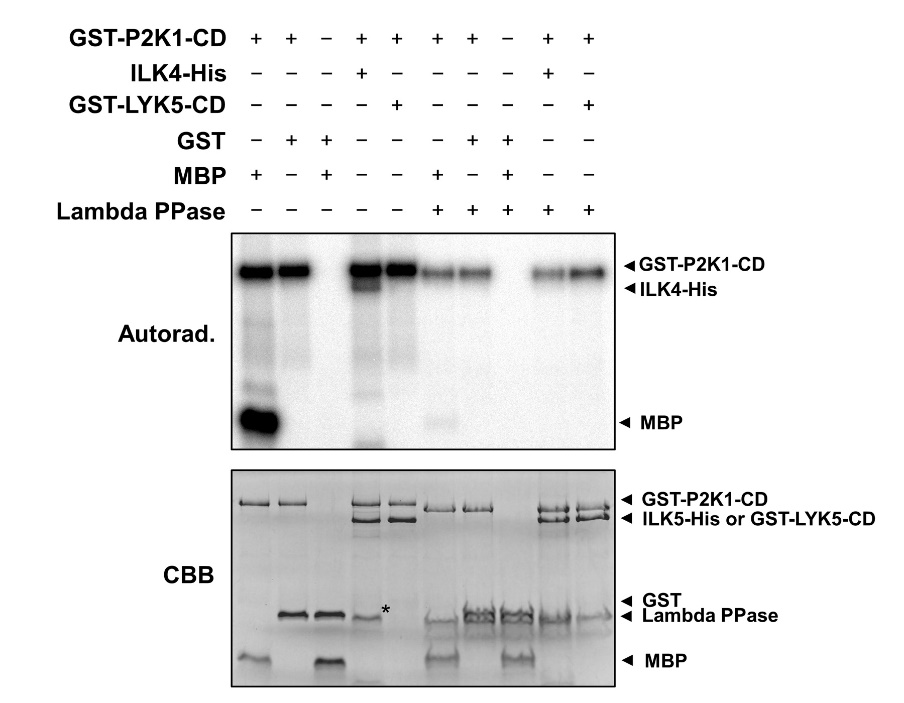


Supplemental Fig. S3. Phosphorylation of ILK4 was confirmed by treatment with lambda protein phosphatase (Lambda PPase), which releases phosphate groups from phosphorylated serine, threonine, and tyrosine residues. Auto- and trans-phosphorylation events were identified through the incorporation of [γ-32P] ATP. Myelin Basic Protein (MBP) was used as a universal substrate, and GST-LYK5-CD was used as a negative control. Protein loading was visualized with Coomassie Brilliant Blue (CBB) staining. Asterisk (*) represents a non-specific protein. These experiments were conducted at least twice and consistently yielded similar results.


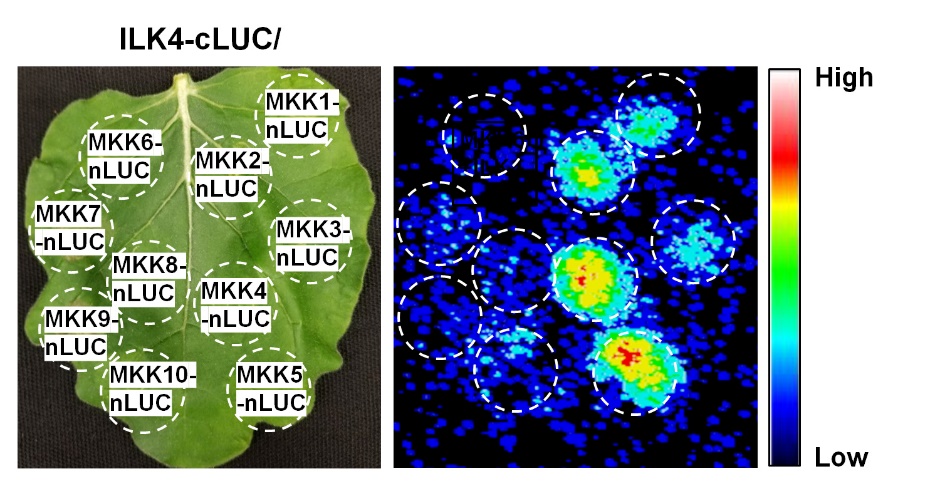


Supplemental Fig. S4. ILK4 interacts with MKKs. The interaction between ILK4 and MKK family members was assessed via a split-LCI assay. Dotted circles mark the regions of interest corresponding to the infiltrated areas in *N. benthamiana* leaves.


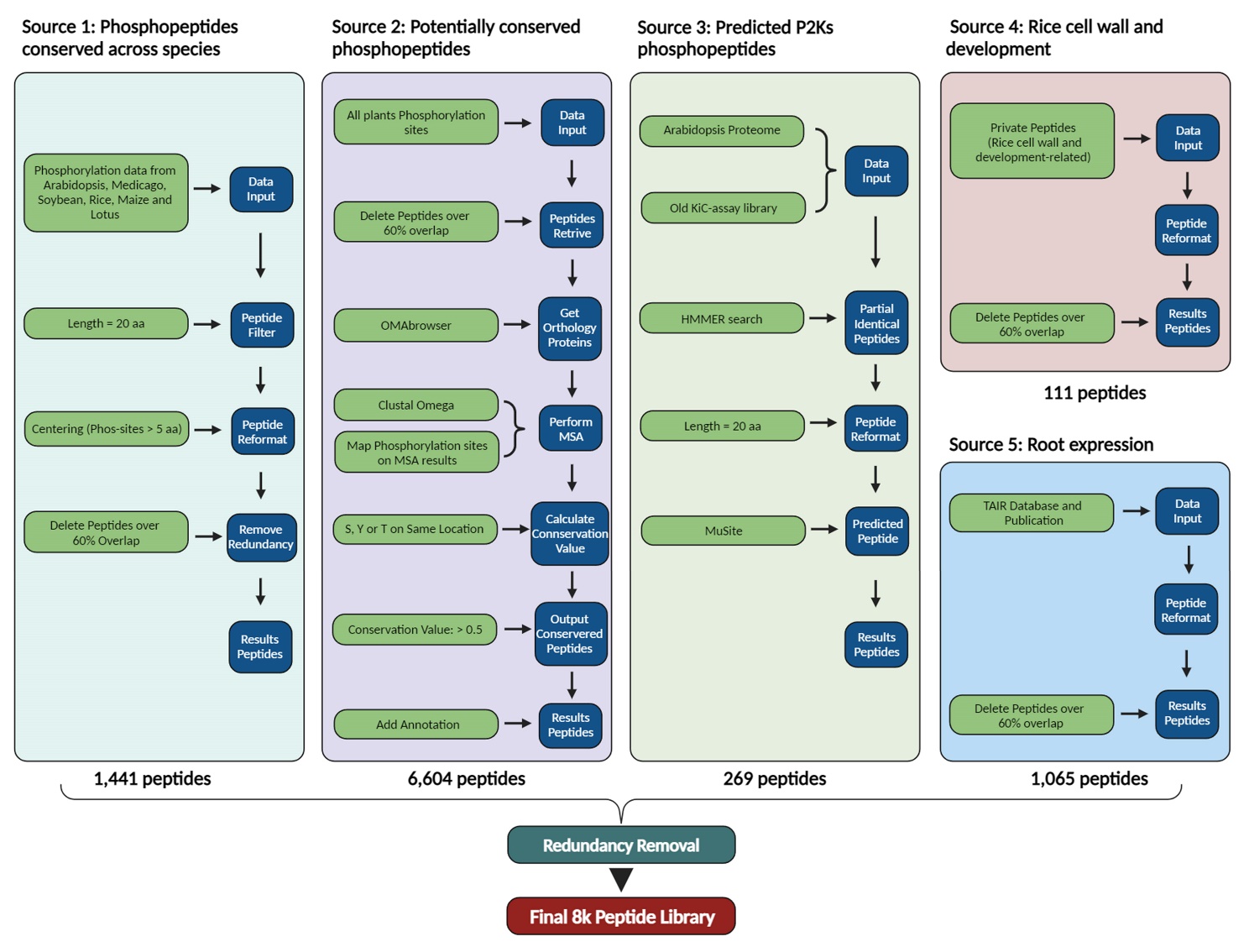
.

Supplemental Fig. S5. 8k peptide library design pipeline for the expanded KiC assay. The design generated five lists based on five different data sources or methods enriched for phosphopeptides either found in other species or having potential for conservation based on sequence similarity. Source 1: Phosphopeptides conserved across species; Source 2: Potentially conserved phosphorylation sites across plant species; Source 3: Predicted phosphorylation sites catalyzed by P2K1 and P2K2 kinase proteins; Source 4: known phosphorylation sites in the rice cell wall and development-related genes. Source 5: Known root expression-related genes. This figure was created using BioRender (https://biorender.com/).


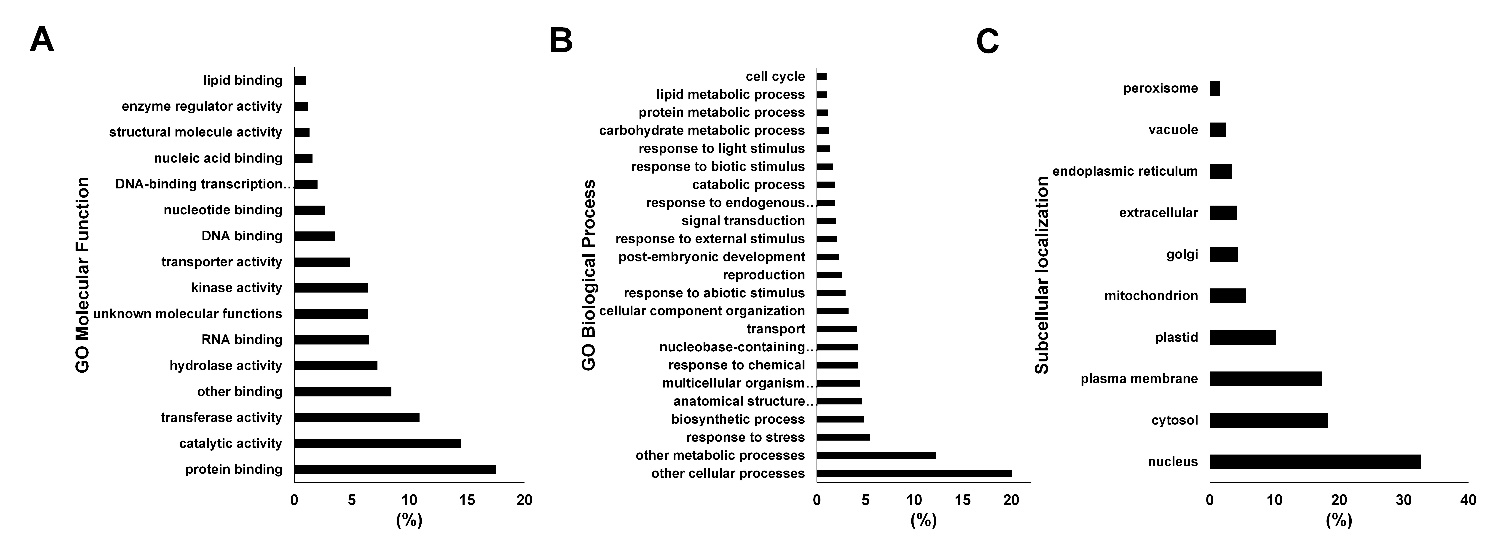


Supplemental Fig. S6. Profile and conserved sequences of the 8k Peptide Library. *A* and *B*, Functional categorization by annotation for GO molecular function (A) and GO biological process (B) among the 8k peptide library. *C,* Analysis of subcellular localization of the 8k peptide library using SUBA5 software.


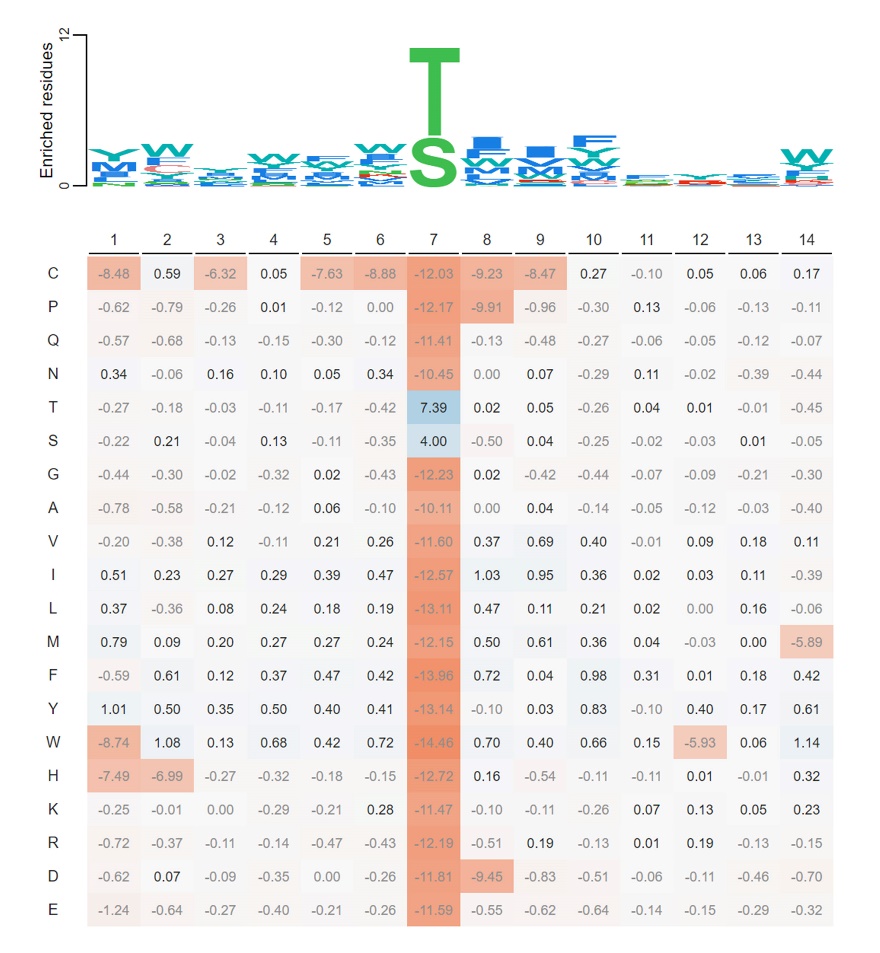


Supplemental Fig. S7. Analysis of conserved sequences in phosphorylated peptides by the P2K1 kinase domain. This figure illustrates the PSSM analysis by using PSMMSearch [*] for P2K1 substrate motif domain. The sequence logo above the heatmap provides a graphical representation of the protein motif's conservation and specificity. The heatmap, using a color gradient from blue (enriched) to red (depleted), represents the PSSM scores calculated for each amino acid at the corresponding motif positions, indicating the likelihood of amino acid occurrence relative to a background distribution. Enriched positions suggest a conserved role in the protein's function or structure, while depleted scores may indicate evolutionary constraints or reduced functional importance.


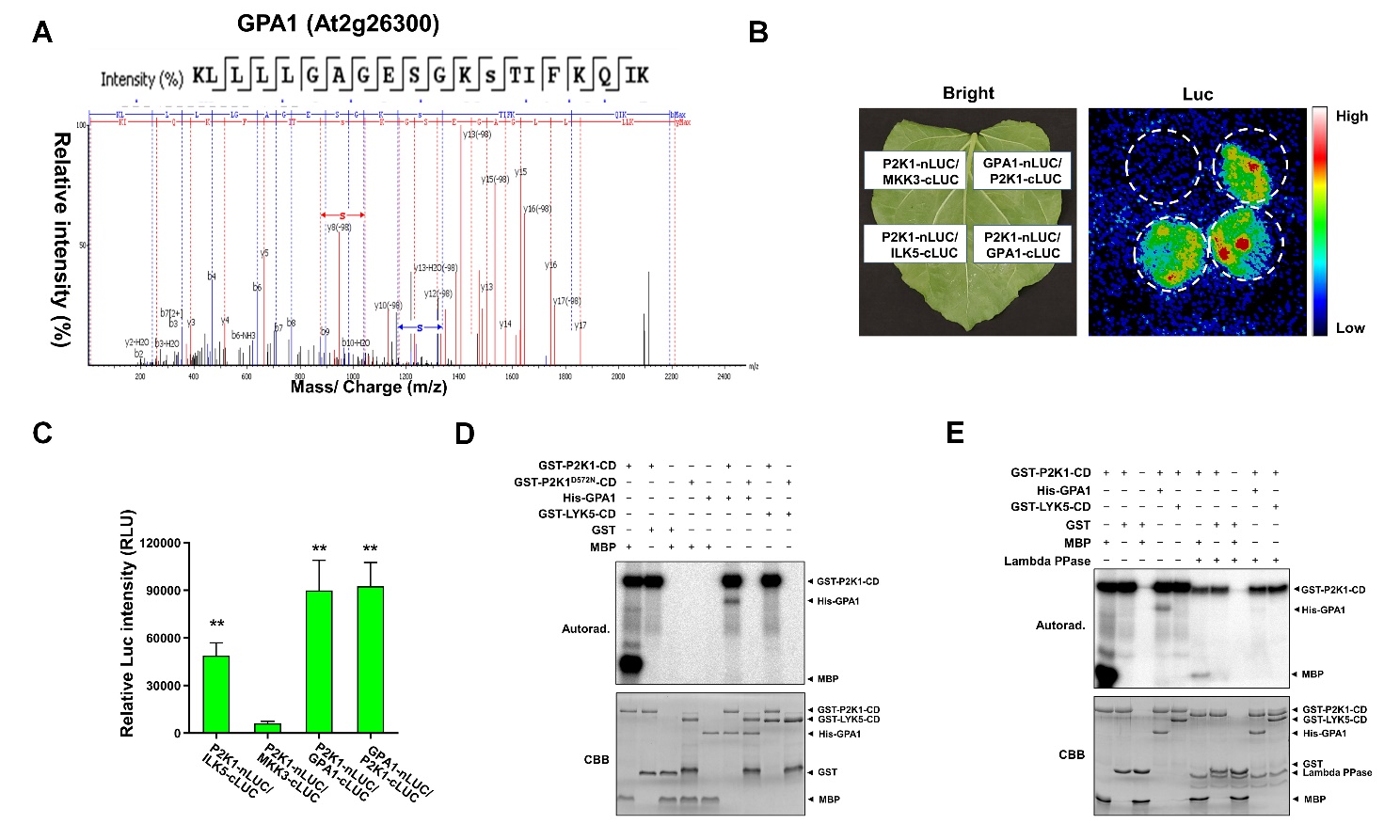


Supplemental Fig. S8. GPA1 was identified as a P2K1 substrate by the KiC assay using the 8k peptide library. *A*, Spectrum of phosphopeptide of GPA1. *B*, The interaction between P2K1 and GPA1 was assessed via a split-LCI assay. Dotted circles mark the regions of interest corresponding to the infiltrated areas in *N. benthamiana* leaves. ILK5-cLUC and MKK3-cLUC proteins were used as a positive and a negative control, respectively. *C*, The P2K1-GPA1 interaction signal intensities were quantified. Images captured during the experiment were analyzed using the C-vision/Im32 software to measure luciferase signal intensities. The data were then analyzed using GraphPad Prism 8. The results are expressed as the mean ± SEM, with 4 biological replicates. Statistical significance was determined via unpaired two-tailed Student’s *t-*test, with levels of significance indicated as follows: *****p* < 0.0001, ****p* < 0.001, ***p* < 0.01, **p* < 0.05. The *p*-values reflect the significance in comparison to the MKK3-cLUC control. *D*, GST-P2K1-CD protein directly phosphorylates His-GPA1, whereas GST-LYK5-CD is not phosphorylated *in vitro*. Bacterial recombinant His-GPA1 protein was incubated with GST-tagged P2K1 cytosolic domain (GST-P2K1-CD, GST-P2K1^D572N^-CD; a kinase-dead version), or GST. *E*, Phosphorylation of GPA1 was confirmed by treatment with lambda protein phosphatase (Lambda PPase), which releases phosphate groups from phosphorylated serine, threonine, and tyrosine residues. Protein loading was visualized with Coomassie Brilliant Blue (CBB) staining.


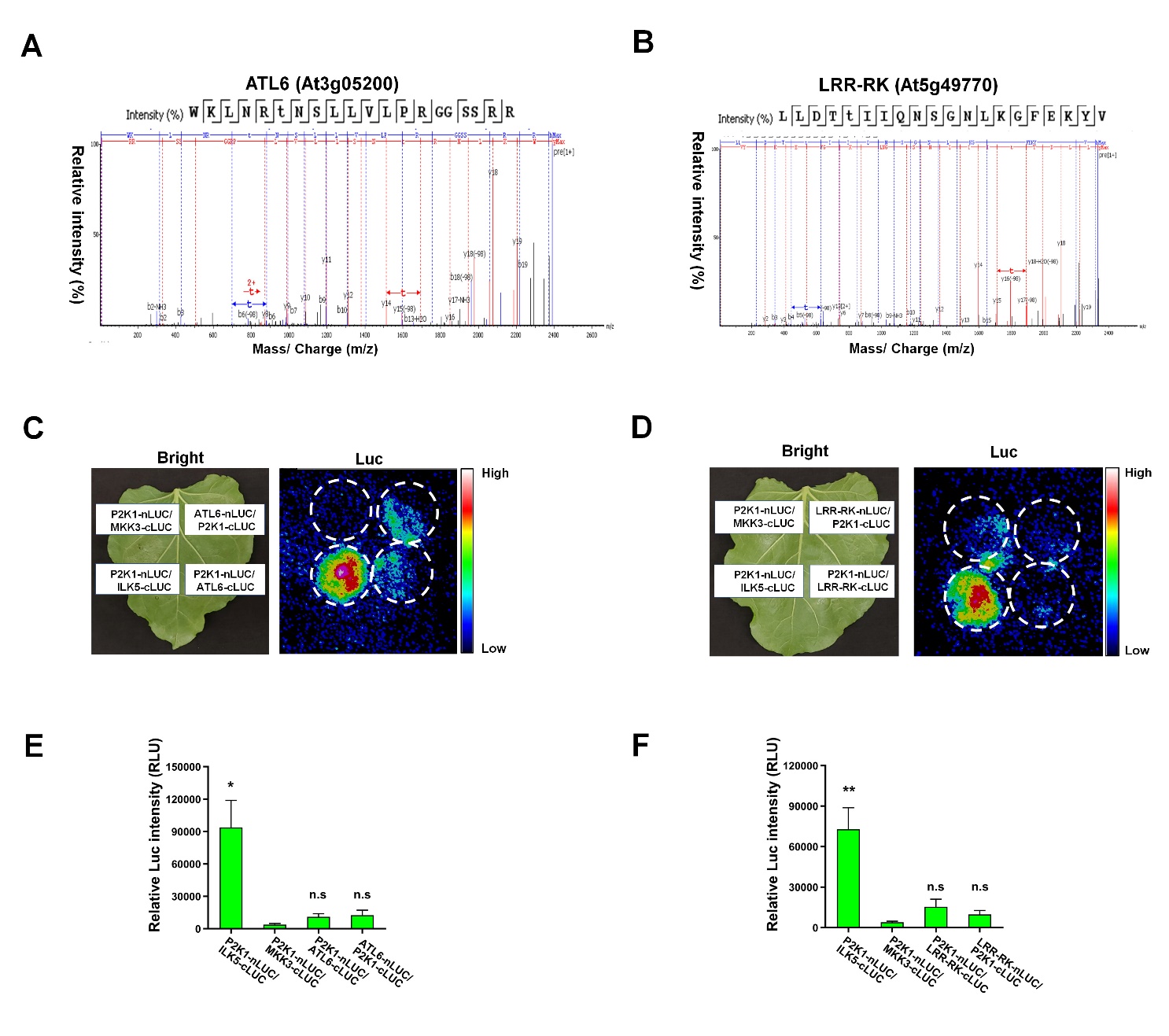


Supplemental Fig. S9. ATL6 and LRR-RK did not show protein-protein interaction with P2K1 in the split-LCI assay. *A* and *B*, Spectrum of phosphopeptide of ATL6 and LRR-RK proteins. *C and D*, The interaction between P2K1 and ATL6 or LRR-RK was assessed via an LCI assay. The assay was conducted in *N. benthamiana* leaves, where P2K1-nLUC and either ATL6-cLUC or LRR-RK were co-infiltrated using *GV3101* agrobacterium. Dotted circles mark the regions of interest corresponding to the infiltrated areas in *N. benthamiana* leaves. ILK5-cLUC and MKK3-cLUC proteins were employed as positive and negative controls, respectively. *E* and *F,* The P2K1-ATL6 or -LRR-RK interaction signal intensities were quantified. Images captured during the experiment were analyzed using the C-vision/Im32 software to measure luciferase signal intensities. The data were subsequently subjected to analysis using GraphPad Prism 8. The results are expressed as the mean ± SEM, with 4 biological replicates. Statistical significance was determined via an unpaired two-tailed Student’s *t-*test, with levels of significance indicated as follows: *****p* < 0.0001, ****p* < 0.001, ***p* < 0.01, **p* < 0.05. The *p*-values reflect the significance in comparison to the MKK3-cLUC control. n.s. represents no significant difference.
